# Genetic variation and inheritance of phytosterol and oil content in a doubled haploid population derived from the winter oilseed rape Sansibar × Oase cross

**DOI:** 10.1007/s00122-015-2621-y

**Published:** 2015-10-30

**Authors:** Lishia Teh, Christian Möllers

**Affiliations:** Department of Crop Sciences, Georg-August-Universität Göttingen, Von-Siebold-Str. 8, 37075 Göttingen, Germany

**Keywords:** *Brassica napus*, Phytosterols, Oil content, Fatty acids, QTL mapping

## Abstract

*****Key message***:**

**Identification of QTL for phytosterol content, oil content, fatty acids content, protein content of defatted meal, and seed weight by multiple interval mapping in a*Brassica napus*DH population.**

**Abstract:**

Phytosterols are minor seed constituents in oilseed rape which have recently drawn wide-interest from the food and nutrition industry due to their health benefit in lowering LDL cholesterol in humans. To understand the genetic basis of phytosterol content and its relationship with other seed quality traits in oilseed rape, QTL mapping was performed in a segregating DH population derived from the cross of two winter oilseed rape varieties, Sansibar and Oase, termed SODH population. Both parental lines are of canola quality which differ in phytosterol and oil content in seed. A genetic map was constructed for SODH population based on a total of 1638 markers organized in 23 linkage groups and covering a map length of 2350 cM with a mean marker interval of 2.0 cM. The SODH population and the parental lines were cultivated at six environments in Europe and were phenotyped for phytosterol content, oil content, fatty acids content, protein content of the defatted meal, and seed weight. Multiple interval mapping identified between one and six QTL for nine phytosterol traits, between two and six QTL for four fatty acids, five QTL for oil content, four QTL for protein content of defatted meal, and three QTL for seed weight. Colocalizations of QTL for different traits were more frequently observed than individual isolated QTL. Major QTL (*R*^2^ ≥ 25 %) were all located in the A genome, and the possible candidate genes were investigated by physical localization of the QTL to the reference genome sequence of *Brassica rapa*.

**Electronic supplementary material:**

The online version of this article (doi:10.1007/s00122-015-2621-y) contains supplementary material, which is available to authorized users.

## Introduction

Oilseed rape (*Brassica napus* L.; genome AACC, 2*n* = 38) is the world’s third-leading source of vegetable oil for human nutrition and industrial products. Almost all of the oilseed rape cultivation is “double low” or “canola” quality” with low content of erucic acid in the oil and glucosinolates in the seeds (Friedt and Snowdon 2010). While oilseed rape breeding has achieved remarkable success over the past few decades, there is still much to learn about the genes regulating seed oil content and quality traits. Increasing concerns about rapid population growth, demands for improved nutritional oil, and expansion of biofuel production have also led to the call for further enhancement in quantity and quality of seed oil. In the case of a complex trait like seed oil content, the number of QTL as reported by numerous studies varied between 3 and 27 QTL and were found distributed among 17 of the 19 chromosomes in *B. napus* (Rahman et al. 2013). In addition, these QTL individually explained between 2 and 10 % of the phenotypic variance while the additive effect ranged from 0.2 % to more than 1.0 % (Rahman et al. 2013). Therefore, increasing oil content through breeding would have to rely on progressive stacking of positive alleles. Besides considering the environmental influence on the QTL, identifying the underlying candidate genes as well as recognizing the pleiotropic effect or correlation between traits would greatly increase the efficiency of breeding. Several studies have shown that oil content is influenced by the fatty acid composition or vice versa (Ecke et al. 1995; Möllers and Schierholt 2002; Hobbs et al. 2004; Zheng et al. 2008). Since triacylglycerols constitute about 90 % of the oil, the fatty acid composition which represents the overall composition of the triacylglycerols is an important quality parameter determining the value and suitability of the oil for nutritional or industrial applications. Although the canola quality oilseed rape possesses a nearly ideal fatty acid profile, there is still room for improvement on the thermal stability of oil by further increasing oleic acid and reducing the polyunsaturated fatty acids content.

Recently, some minor salutary oil constituents such as carotenoids (Shewmaker et al. 1999; Yu et al. 2008; Wei et al. 2010), phytosterols (Amar et al. 2008b), and tocopherols (Marwede et al. 2005; Fritsche et al. 2012; Wang et al. 2012b) have also drawn the attention among plant breeders and researchers to study and improve the content and composition due to their conferred health-benefiting properties. Phytosterols are widely known for their cholesterol lowering properties since 1950s (Peterson 1951; Pollak 1953). An effective dose of 1–3 g day^−1^ leads to reduction between 8 and 15 % in LDL cholesterol (Quilez et al. 2003). Other promising effects include anti-cancer (Woyengo et al. 2009), anti-atherosclerosis (Moghadasian et al. 1997), anti-inflammation (Bouic 2001), and anti-oxidation (Van Rensburg et al. 2000). These health-promoting properties have led to the development of functional foods enriched with phytosterols as bioactive ingredients. A variety of foods fortified with phytosterols, including margarines, mayonnaises, vegetable oils, salad dressings, milk, dairy products, beverages, and snack bars, are now widely available in the market (Berger et al. 2004). The most common sources of phytosterol added to foods are tall oil—a byproduct of the pulping industry that is rich in sitosterol and sitostanol (Jones et al. 1998)—and distillate fraction from vegetable oil refining. While most crude vegetable oils contain about 1–5 g kg^−1^ of phytosterol, corn oil contains about 8–16 g kg^−1^ and oilseed rape oil contains about 5–10 g kg^−1^ (Piironen et al. 2000). The high amount of phytosterol in oilseed rape means that it may serve as valuable base stock for the health and nutrition industry.

**Fig. 1:**
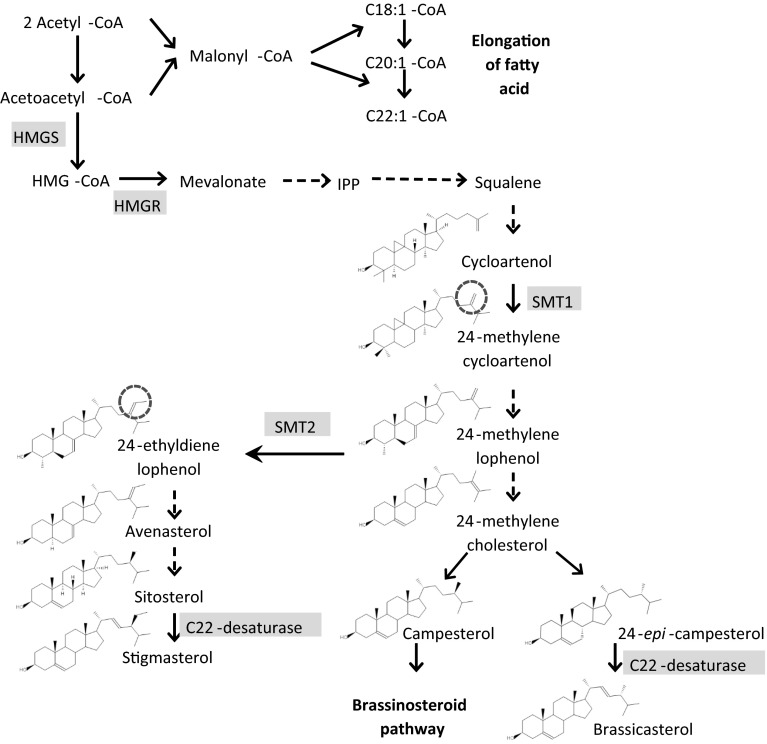
Simplified phytosterol biosynthetic pathway in plants. *Solid* and *dashed arrows* indicate single and multiple biosynthetic steps, respectively. Adapted from Benveniste (2002), Schaller (2003). *HMGS* HMG-CoA synthase, *HMGR* HMG-CoA reductase, *SMT1* C-24 sterol methyltransferase 1, *SMT2* C-24 sterol methyltransferase 2

Phytosterols include a wide variety of molecules that are structurally similar to cholesterol. The structural variations of phytosterols arise from different number of carbon atoms on C-24 in the side chain as well as the number and position of double bonds in the tetracyclic skeleton (Fig. 1). In oilseed rape, the phytosterol profile consists mainly of sitosterol, campesterol, brassicasterol, and avenasterol, while cholesterol and stigmasterol occur only in trace amounts (Appelqvist et al. 1981). Brassicasterol is a characteristic sterol of *Brassicaceae* species and in oilseed rape, it amounts to about 13 % of total phytosterol content. Among the adapted winter oilseed rape populations, modern cultivars with canola quality contain higher amount of total phytosterols than the genetically diverse or resynthesized lines that are of non-canola quality. This observation is due to the close negative correlation between total phytosterol content and erucic acid content (Amar et al. 2008b). In a winter oilseed rape DH population segregating for erucic acid, QTL mapping showed that two of the three QTL identified for total phytosterol content colocalized with two erucic acid genes (Amar et al. 2008a). Based on the fact that cytoplasmic acetyl-CoA is required in the synthesis of both erucic acid and phytosterols, colocalizations of QTL are most likely attributed to pleiotropic effect exerted by the erucic acid genes. To further investigate the inheritance of phytosterols and their relations to other important seed quality traits, a DH population constructed from the two canola quality winter oilseed rape cultivars, Sansibar and Oase, was used in this study. The parental lines were shown to differ with respect to phytosterol and oil content based on a previous screening (Amar et al. 2009). It was anticipated that this DH population, which does not segregate for erucic acid, may have greater power for detection of QTL with novel alleles for phytosterol content than previous studies. The main objectives of this study were (1) to identify QTL for phytosterol content, fatty acids, oil content, protein content of defatted meal, and seed weight; (2) to investigate the correlation between the analyzed traits; and (3) to inspect for possible candidate genes underlying the major QTL.

## Materials and methods

### Plant material

The experimental population consisted of 226 F1 microspore-derived DH lines derived from the Sansibar × Oase cross. The two parental lines were among the 27 canola quality winter oilseed rape cultivars analyzed by Amar et al. (2009) and were chosen due to their contrasting total phytosterol content and oil content in seed; Sansibar had the highest total phytosterol content (~480 mg 100 g^−1^ seed) and lowest oil content (43 %), while Oase had the lowest total phytosterol content (~360 mg 100 g^−1^ seed) and highest oil content (46 %). The DH population was developed in the Division of Plant Breeding at Georg-August-Universität Göttingen and was named as SODH population.

### Field experiments

The SODH population and the parental lines were cultivated in six environments: two environments at Göttingen, Germany during growing seasons 2009/2011 and 2010/2011; one environment at Einbeck, Germany during growing season 2010/2011 by KWS Saat AG; one environment at Asendorf, Germany during growing season 2011/2012 by Deutsche Saatveredelung (DSV) AG; and two environments at Svalöv, Sweden during growing seasons 2010/2011 and 2011/2012 by Lantmännen SW Seed. The field trials were carried out in small plots in a complete randomized design without replication. Seeds of ten open pollinated plants from each line were harvested and bulked for analyses.

### Molecular markers

Genomic DNA of the SODH population and their parental lines were isolated from young leaves of 4–5 week-old greenhouse-grown seedlings using Nucleon PhytoPure plant extraction kits (GE Healthcare, Illustra*™*) according to manufacturer’s instructions. DNA was quantified using Bio-Rad Fluorescent DNA Quantification Kit (Bio-Rad Laboratories CA, USA).

#### Simple sequence repeats (SSR) and amplified fragment length polymorphism (AFLP) markers

SSR analysis was carried out following the M13-tailing PCR technique (Schuelke 2000). PCR reactions were performed in 96-well PCR plates with a volume of 20 μl per reaction, containing 25 ng of genomic DNA, 0.05 μM of forward primer with a M13 tail of 19 bp at the 5′ end, 0.05 μM of reverse primer, 0.05 μM of M-13 primer, 2.5 mM MgCl_2_, 0.2 mM of each dNTP, 1× PCR buffer, and 1 U of *Taq* DNA polymerase. A two-step touchdown PCR program was performed in a Biometra T1 Thermocycler (Biometra GmbH, Göttingen, Germany): 95 °C for 2 min; 5 cycles of 95 °C for 45 s; 68 °C (−2 °C/cycle) for 5 min, 72 °C for 1 min; 5 cycles of 95 °C for 45 s, 58 °C (−2 °C/cycle) for 1 min, 72 °C for 1 min; 27 cycles of 95 °C for 45 s, 47 °C for 30 s and 72 °C for 1 min; and 72 °C for 10 min. A total of 350 primer pairs obtained from various sources were screened for polymorphisms between the parents. The SSR primer pairs prefixed with “BRA” and “CB” were developed by Celera AgGen consortium, and prefixed with “MR” and “MD” were developed by Division of Plant Breeding at Georg-August-Universität Göttingen. AFLP analysis was performed by adapting the method described by Vos et al. (1995). A total of 16 primer combinations made up from 8 *EcoRI* fluorescence-labeled primers and 4 *MseI* primers were used: E32M48, E37M50, E36M51, E36M59, E39M48, E38M50, E37M51, E37M59, E44M48, E40M50, E38M51, E38M59, E45M48, E44M50, E44M51, and E44M59. The PCR products of AFLP and SSR were separated on the ABI PRISM 3100 genetic analyzer (Applied Biosystems) with GeneScan-500 ROX size standard (Applied Biosystems) using 36 cm capillary arrays. The results were analyzed with GeneScan software version 3.7 (Applied Biosystems) and scored using Genotyper software version 3.7 NT (Applied Biosystems).

#### Single nucleotide polymorphism (SNP) markers

A total of 125 polymorphic SNP, designated with prefix “ra” were genotyped by the breeding company KWS Saat AG and were kindly provided to us for map construction.

#### Diversity arrays technology (DArT) and Silico-DArT markers

The SODH population was genotyped with the *B.* *napus* v1.0 DArT microarray comprising 3072 markers, designated with the prefix “brPb.” A subset of 183 lines from the SODH population was genotyped with 4787 Silico-DArT markers (www.diversityarrays.com/dart-application-dartseq-data-types), designated with the suffix “| F |0.” Genotyping with DArT and Silico-DArT markers was performed by Diversity Array Technology Pty Ltd, Yarralumla, Australia. The sequences for DArT markers were retrieved from http://www.diversityarrays.com/dart-map-sequences while the sequences for Silico-DArT clones were provided by Diversity Array Technology Pty Ltd, Yarralumla, Australia.

#### Kompetitive allele specific PCR (KASP) markers

From the Illumina Infinium Brassica 60 K SNP array, a subset of 32 markers that were polymorphic between the parental lines, Sansibar and Oase, were selected for KASP genotyping (Trait Genetics GmbH). Of the 32 markers, 13 were physically closely linked to promising candidate genes for phytosterol biosynthesis and 19 were associated with oil content in SGDH14 × Express617 DH population (Nina Behnke, personal communication). The sequences for SNP markers were provided by Isobel Parkin (AAFC, Saskatoon, Canada). The physical positions were based on reference genome of *B. rapa* v1.5 genome database (BRAD; http://www.brassicadb.org/brad/) (Wang et al. 2011) and *B. oleracea* v1.0 genome database (Bolbase; http://www.ocri-genomics.org/bolbase/). KASP markers were prefixed with “Bn-.”

#### Candidate gene-based markers

Five candidate genes involved in the regulation of phytosterol synthesis and one candidate gene involved in the triacylglycerol synthesis were selected to develop candidate gene-based markers. The details of the candidate genes are described in Supplementary Table 1 with the aid of the phytosterol biosynthetic pathway depicted in Fig. 1. The approach involved first designing a locus-specific marker to differentiate between homologs based on locus-specific SNP, followed by sequencing of the amplicons to screen for allelic SNP between the parental lines. If an allelic SNP was found, an allele-specific marker was developed for the pertaining homolog. Due to the limited number of locus-specific SNP and a lack of polymorphisms between the parental lines, only four candidate gene-based markers were developed: HMG1A07-O1 for *3*-*hydroxy*-*3*-*methylglutaryl*-*CoA reductase 1* (*HMG1*) and HMG2A10-2 for *hydroxy*-*3*-*methylglutaryl*-*CoA reductase 2* (HMG2), and D120E-3 and Dx-3 for *diacylglycerol acyltransferase 1* (*DGAT1*). The *DGAT1* primer pairs were kindly provided by Dr. Renate Schmidt from IPK Gatersleben. Primer sequence of candidate gene-based markers are listed in Supplementary Table 2.

### Linkage map of SODH population

Linkage map was constructed using MAPMAKER/EXP 3.0 (Lincoln et al. 1992) with the aid of a purpose-built Perl script (unpublished; Wolfgang Ecke, personal communication) that automates the mapping process. Segregation of each marker was tested by *χ*^2^ analysis (*P* = 0.05) to assess the goodness-of-fit for the expected segregation ratio (1:1). Markers which were significantly deviating from 1:1 segregation ratio were regarded as skewed segregated markers while markers which were not significantly different from 3:1 or beyond were defined as strongly skewed segregated markers. Markers with strongly skewed segregation were initially excluded for map construction and were attempted for mapping after the initial map was built.

Markers were assigned to linkage groups to construct a core map by the “group” command with the minimum LOD score parameter set to 4 and the maximum distance parameter set to 35 cM. The most probable marker order within each group was determined by the command “order” and the resulting high-fidelity map was built upon by adding markers using the command “try.” Markers that showed more than the predetermined numbers of crossovers were excluded in the high-fidelity map. Markers that were not supported by a LOD score of 3 in the high-fidelity map were placed at their most likely position in the linkage group. Following this, the “ripple” command was used to find the optimal marker order in the linkage groups. Genetic distances between loci were calculated using the Kosambi mapping function (Kosambi 1944). The resulting map consisted of high-fidelity markers which are supported by a LOD score of at least three and placed markers which are supported with LOD score of less than three.

The map was further optimized by constructing each linkage group 200 times with a random subset of five highly informative markers according to MAPMAKER/EXP3.0 command order to obtain the possible variant of a high-fidelity map. The optimal variant was selected to have as many markers as possible, as few double crossover as possible, and that the markers were as evenly distributed as possible.

The map was aligned with common marker loci on established genetic maps based on SSR (Piquemal et al. 2005; Radoev et al. 2008; Sharpe and Lydiate, unpublished data), DArT (Raman et al. 2013), and SNP (KWS Saat AG, unpublished data). Linkage groups were named according to the nomenclature of Parkin et al. (1995) as A01–A10 and C01–C09.

For QTL mapping purpose, a subset of markers were selected from the high-fidelity markers on the basis that the distance between adjacent markers was about 5–10 cM. The term framework map was used to refer to the map used for QTL mapping.

### Phenotypic analysis

#### Phytosterols

Phytosterol content was analyzed by adapting the protocol of Amar et al. (2008b) and Fernández-Cuesta et al. (2012), following a direct alkaline hydrolysis method which involves three major steps: alkaline hydrolysis (saponification), extraction of the non-saponifiable matter, and derivatization of the sterols to trimethylsilyl (TMS)-ether derivatives. The main advantage of using this method is that it bypasses the lipid extraction step, facilitating large number of seed samples to be analyzed more economically. The downside of this method is that alkaline hydrolysis could only quantify free sterols and steryl esters, but not steryl glycosides. The hydrolysis of acetal bond between phytosterol and the carbohydrate moiety requires acidic condition which may be destructive to the compound and laborious for routine analysis. Hence, it is possible that the present analysis would underestimate the total phytosterol concentration in the seed sample.

For each sample analysis, 200 mg of seed was weighed and placed in a polypropylene tube. Two milliliter of 2 % potassium hydroxide (Carl Roth, Germany) in ethanol (w/v) was added for alkaline hydrolysis, followed by 200 μl of 2 % cholesterol (99 % purity, Sigma-Aldrich, Germany) in hexane-ethanol (3:2) solution, used as an internal standard to quantify phytosterol content. By placing one stainless steel rod (1.1 cm length; 0.4 cm diameter) in each tube, seeds were crushed and homogenized using a custom-built vertical homogenizer (Institute of Applied Plant Nutrition, Georg-August-Universität Göttingen) for 3 min at a speed deemed sufficient to homogenize the seeds. The tubes were subsequently incubated for 15 min at 80 °C in a water bath and cooled at room temperature for 30 min. To extract the phytosterols, 1.0 ml of hexane and 1.5 ml of distilled water were added, briefly vortexed, and centrifuged for 10 min at 4000 rpm. The upper hexane layer was transferred to a new tube and left over night on a hot plate at 37.5 °C to evaporate. The residue obtained after evaporation was dissolved with 80 μl hexane and derivatized with 20 μl of silylating agent, composed of hexamethyldisilazane (Fluka analytical):trimethylchlorosilane (Sigma-Aldrich purum >98 %; GC grade) 3:1. The solution was pipetted into a GC vial, capped, and incubated at room temperature for 20 min. To settle the precipitate, the derivatized samples were centrifuged for 10 min at 3000 rpm prior to GC analysis.

Analysis of derivatized sterols was performed using capillary gas–liquid chromatograph (Chrompack CP-9003), equipped with autosampler, split injector (320 °C; injection volume of 3 μl with a split ratio of 100:1), and flame ionization detector 320 °C, with fused silica capillary column of medium polarity (SE-54, 50 m long, 0.1 μm film thickness, 0.25 mm i.d. coated with 5 %-phenyl-1 %-vinyl-methylpolysiloxane) (IVA Analysentechnik, Meerbusch, Germany). Hydrogen (carrier gas) pressure was set at 150 kPa. Initial oven temperature was set at 240 °C with an increment of 5 °C per min to final oven temperature at 275 °C and held for 20 min. Total analytical time was 25 min.

Phytosterol content was expressed as mg 100 g^−1^ seed The phytosterol traits evaluated in this study include contents of brassicasterol, campesterol, sitosterol, avenasterol, total phytosterol, 24-methyl sterol, 24-ethyl sterol and campesterol to sitosterol ratio, and 24-methyl to 24-ethyl sterol ratio. Total phytosterol content was calculated as the sum of brassicasterol, campesterol, sitosterol, and avenasterol contents. 24-Methyl sterol was calculated as the sum of brassicasterol and campesterol contents. 24-Ethyl sterol was calculated as the sum of sitosterol and avenasterol contents.

#### Fatty acids

Fatty acid composition was analyzed by gas chromatography using a method adapted from Thies (1971). Approximately 200 mg of seed, 1 ml of Na-methylate-methanol (0.5 mol l^−1^), and one stainless steel rod (1.1 cm length; 0.4 cm diameter) were added in a propylene tube. The seeds were then homogenized using a custom-built vertical homogenizer (Institute of Applied Plant Nutrition, Georg-August-Universität Göttingen) for 3 min. Following incubation for 20 min at room temperature, 300 μl isooctane and 100 μl 5 % NaHSO_4_ in water were added, briefly vortexed, and centrifuged for 3 min at 4000 rpm. About 200 μl of the upper phase was pipetted into a GC vial and 3 μl was injected into a gas chromatograph (Thermo Trace GC Ultra), equipped with autosampler, split injector (split ratio 70:1), flame ionization detector (320 °C), and capillary FFAP-phase (0.25 mm × 25 m; Macherey & Nagel). Hydrogen (carrier gas) pressure was set at 100 kPa. Oven temperature was set at 210 °C. Total analytical time was 6 min.

The fatty acid content reported in this study include palmitic acid (C16:0), oleic acid (C18:1), linoleic acid (C18:2), and linolenic acid (C18:3), expressed as percentage of total fatty acids (including other minor fatty acids) in mature seeds.

#### Oil and protein content of defatted meal

Oil and protein content in seeds were estimated by NIRS using calibration raps2012.eqa provided by VDLUFA Qualitätssicherung NIRS GmbH (Teichstr. 35, D-34130 Kassel, http://h1976726.stratoserver.net/cms, accessed September 24, 2015). Oil content and protein content of defatted meal were expressed as a percentage of seed dry matter content at 9 % moisture.

Protein content of defatted meal was calculated by using the estimated seed oil content and seed protein content obtained from the NIRS prediction as follows:

$${\%\,\text{Protein of defatted meal}} = \frac{{\%\, {\text{Seed}}\;{\text{protein}}}}{{100 - \%\,{\text{Seed}}\;{\text{oil}}}} \times 100\,\%.$$

#### Seed weight

Thousand seed weight was obtained from weight conversion of 500 seeds. The seeds were counted using a seed counter (Model:Contador, Pfeuffer GmbH, D-97318 Kitzingen, http://www.pfeuffer.com).

### Statistical analysis

Variance components, heritability, and means were estimated using PLABSTAT software version 3A (Utz 2011). The model implemented in ANOVA analysis was as follows:

$$Y_{ij} = \mu + g_{i} + e_{j} + \varepsilon_{ij},$$

where $Y_{ij}$ is the trait value of the *i*th genotype in the *j*th environment, $\mu$ is the general mean, $g_{i}$ is the effect of *i*th genotype, $e_{j}$ is the effect of *j*th environment, and $\varepsilon_{ij}$ is the random error mean of the *i*th genotype in the *j*th environment confounded with residual error and genotype × environment interaction. The genotype was treated as fixed effect, whereas environment was treated as random effect.

Broad-sense heritability $\left( {\hat{h}^{2} } \right)$ was estimated as follows:

$$\hat{h}^{2} = \frac{{\hat{\sigma }_{\text{G}}^{2} }}{{\hat{\sigma }_{\text{G}}^{2} + {\raise0.7ex\hbox{${\hat{\sigma }_{\varepsilon }^{2} }$} \!\mathord{\left/ {\vphantom {{\hat{\sigma }_{\varepsilon }^{2} } {n_{\text{e}} }}}\right.\kern-0pt} \!\lower0.7ex\hbox{${n_{\text{e}} }$}}}},$$

where $\hat{\sigma }_{\text{G}}^{2}$ and $\hat{\sigma }_{\text{E}}^{2}$ are variance components for genotype and random error; *n*_e_ refers to number of environment. Mean values across all environments were used to calculate Spearman’s rank correlation coefficients between traits.

### QTL mapping

QTL detection was performed with WinQTL Cartographer software ver. 2.5 (Wang et al. 2012a) using means of phenotypic data obtained from 6 environments and a framework map consisting of 273 markers. QTL were initially detected with composite interval mapping (CIM) using the default model (model 6) that selects certain markers as control markers by using additional parameters. For each trait, the LOD significance threshold (*α* = −0.05) were estimated by 1000 permutation tests. Five markers selected by a forward and backward regression method were used as cofactors. CIM tests were performed at 1-cM steps with a 10-cM window size. Peaks were treated as separate QTL when the distance is more than 5 cM and the minimum LOD value exceeds one between any two adjacent peaks.

Subsequently, multiple interval mapping (MIM) was performed to refine the QTL positions, to search for more QTL, and to investigate epistatic effects among the detected QTL (Kao et al. 1999). The MIM model was built upon a priori model from CIM analysis and progressively refined using the BIC-M2 = 2ln(*n*) criterion. QTL positions that did not remain significant when fitted with others were then dropped from the model. QTL effects and their percentage of phenotypic variance explained by individual and all the QTL were estimated with the final model fitted in MIM. A one-LOD drop from the peak position was used as a confidence interval for each QTL.

### *In silico* mapping of sequence-informative markers on the *B. napus* genome

Sequences of DArT, silico-DArT, SSR, and KASP markers were used to search in the *B. napus* Darmor-*bzh* genome sequence assembly (Chalhoub et al. 2014) using the nucleotide MEGABLAST algorithm. The word size was set at 28 and the cutoff e-value was set at 1e-10. When multiple hits were obtained, the physical marker position was predicted based on the alignment with the genetic map.

### Identification of possible candidate genes for major QTL

Based on known key regulatory genes from the literature, an attempt was made to investigate whether the predicted genes were in fact underlying the major QTL. Since all the major QTL were located in the A genome, sequences of predicted *Arabidopsis* genes were searched in both *B.* *rapa* Chiifu (Wang et al. 2011) and *B.* *napus* Darmor-*bzh* (Chalhoub et al. 2014) genome sequence assemblies. Likewise, markers within the major QTL region were searched against both *B. rapa* and *B. napus* genome sequence assemblies using the nucleotide MEGABLAST algorithm with word size of 28 and cutoff e-value at 1e-10.

## Results

### Polymorphism of molecular markers and linkage map development of SODH population

Different types of molecular markers were used in the construction of the genetic map for the SODH population: AFLP, SSR, DArT, Silicor-DArT, SNP, KASP, and candidate gene-based markers. With 16 AFLP primer combinations, a total of 75 polymorphic markers could be scored in the SODH population. Of the 350 SSR primer pairs screened, 23 (0.07 %) were found polymorphic between the parents and exhibited clear and unambiguous amplification. Seven of the 23 SSR primer pairs amplified more than one polymorphic locus, resulting in 32 SSR loci. Approximately 13 % (407/3072) of DArT and 42 % (2005/4787) of silico-DArT markers were polymorphic between the parents.

After removal of markers with a minor allele frequency of less than 10 %, a total of 2555 marker loci were available for map construction. The resulting linkage map for SODH population has 1638 markers mapped onto 23 linkage groups and covered 2350.2 cM with a mean interval distance of 2.0 cM between markers. The unmapped markers were either ambiguously linked to various linkage groups, unlinked, or formed small linkage groups that were excluded for estimation of the linkage map length. About 50 % (457/913) of the unmapped markers showed skewed segregation of which 47 % (217/457) showed strongly skewed segregation. The number of markers, map size, marker density, and mean distance between markers are summarized in Table 1 and the genetic map is shown in Supplementary Fig. 1 and Supplementary Table 3. All linkage groups could be assigned with chromosome names according to the nomenclature of Parkin et al. (1995) as A01–A10 and C01–C09. The 23 linkage groups represented 19 chromosomes in *B. napus*, additional four linkage groups (A08-II, C02-II, C03-II, and C04-II) were formed due to loose or no linkage to their main linkage groups.

**Table 1:** Marker distribution, size, density, and mean distance between markers of each linkage group in the linkage map of SODH population

Linkage group	No. of markers per linkage group								Size (cM)	Marker density (cM^−1^)	Average distance between markers (cM)^b^
	AFLP	CG^a^	DArT	KASP	Silico-DArT	SNP	SSR	Total			
A01	2		6	1	58	4	3	74	98.0	0.76	1.50
A02	3		5		26	3		37	40.7	0.91	1.30
A03	7		19	3	107	15	1	152	224.1	0.68	1.70
A04	2		14		84	5	1	106	194.2	0.55	2.10
A05	2		7	2	63	5	2	81	163.8	0.49	2.20
A06	4		18	2	95	5		124	128.0	0.97	1.20
A07	1	3	9	2	134	15		164	133.7	1.23	0.90
A08	2		9	1	31	2		45	37.8	1.19	1.10
A08-II			1		6			7	9.7	0.72	1.90
A09	3		8	2	49	2	1	65	130.2	0.50	2.50
A10	8		11		107	3		129	94.1	1.37	0.90
C01			4		24	4	1	33	77.5	0.43	2.70
C02			1	1	11	1		14	48.2	0.29	4.40
C02-II	1		1		16	1	1	20	97.9	0.20	5.40
C03	2		5	2	72	3		84	111.3	0.75	1.40
C03-II	3		5	1	45	6	1	61	134.8	0.45	2.40
C04			1		19	2	2	24	79.5	0.30	3.60
C04-II	5		3	4	75	6		93	101.0	0.92	1.20
C05	2		1	1	45	4		53	92.1	0.58	1.90
C06	2		5	1	49	7		64	95.2	0.67	1.60
C07	8		8	2	91	7	5	121	142.4	0.85	1.40
C08	4				27	2		33	52.5	0.63	1.80
C09	1	1	3		44	4	1	54	63.5	0.85	1.20
A	34	3	107	13	760	59	11	984	1254.3	0.79	1.57
C	28	1	37	12	518	47	12	654	1095.9	0.60	2.42
Whole	62	4	144	25	1278	106	23	1638	2350.2	0.70	2.01

^a^CG: candidate gene-based markers

^b^Co-segregating markers are represented as a single marker in the calculation of mean distance between markers

The map has an average density of 0.70 marker per cM with distribution of markers varying from 0.20 to 1.37 cM across the linkage groups (Table 1). The A genome comprised more markers (987) as compared to the C genome (655), with a mean interval distance between markers of 1.6 cM in the A genome and 2.4 cM in the C genome. The number of markers mapped in an individual linkage group ranged from 7 (A08-II) to 164 (A07).

About 44 % of the mapped markers (718) showed significant (*P* = 0.05) segregation distortion with the majority (76 %) of the markers favoring the Sansibar allele. Loci with skewed segregation favoring the Sansibar allele were mostly found on linkage groups A07, A10, C03, and C05; while loci with skewed segregation favoring the Oase allele were clustered mainly on linkage groups A05, C01, C03-II, and C04-II. Three candidate gene-based markers (HMG1A07-O1, HMG2A10-2S, and D120E-3) were mapped on A07 and another (Dx-3) was mapped on C09 (Supplementary Table 3).

A total of 910 sequence-informative markers were physically mapped to the *B. napus* genome sequence. The alignment of the SODH genetic map and the physical map of *B. napus* genome sequence was generally in agreement although some regions showed disruption of colinearity which may suggest chromosomal rearrangements, error in genome sequence assembly, or inaccuracies of the map (Supplementary Fig. 2). The genomic locations of sequence-informative markers and their homology are provided in Supplementary Table 4.

### Phenotypic analysis

Highly significant effects for the genotype and the environment were found for all traits in the SODH population (Table 2). Broad-sense heritability ($\hat{h}^{2}$) estimates were high, ranging from 0.80 to 0.90, indicating that much of the phenotypic variance were genetically determined. The total phytosterol content ranged from 311.2 to 486.9 mg 100 g^−1^ seed, with a mean of 401.9 mg 100 g^−1^ seed (Table 3). Among the four quantified end-products of the sterol pathway, sitosterol was the most prominent sterol, followed by campesterol, brassicasterol, and avenasterol. The 24-ethyl sterol content, which includes sitosterol and avenasterol, was higher than the 24-methyl sterol content, which comprises campesterol and brassicasterol. Between the parents, Sansibar consistently showed a higher phytosterol content than Oase while Oase had a higher 24-methyl to 24-ethyl sterol ratio than Sansibar and only a small difference was observed for the campesterol to sitosterol ratio. The oil content was high in this population, ranging from 41.2 to 48.6 %, with a mean of 46.3 %. Between the parents, Oase had a higher oil content than Sansibar.

**Table 2:** Variance components and heritability of the SODH population (*n* = 226)

Trait	Variance components (σ^2^)			Heritability
	Genotype (G)	Environment (E)	*ε*	$\hat{h}^{2}$
*Phytosterol* $\big( {{\text{mg }}100 {\text{g}}^{ - 1} } {\text{seed}} \big)$				
Brassicasterol	14.28**	5.06**	16.12	0.84
Campesterol	315.99**	160.43**	150.54	0.93
Sitosterol	267.43**	36.23**	310.05	0.84
Avenasterol	48.38**	94.09**	52.44	0.85
Total sterol	1139.02**	706.69**	934.69	0.88
24-methyl sterol	330.13**	188.89**	192.91	0.91
24-ethyl sterol	412.95**	206**	368.78	0.87
Campesterol:sitosterol^a^	89.77**	24.56**	41.65	0.93
24-methyl:24-ethyl sterol^a^	62.9**	8.29**	33.99	0.92
*Other traits*				
C16:0 (%)	0.1**	0.04**	0.07	0.90
C18:1 (%)	2.57**	0.52**	1.48	0.91
C18:2 (%)	1.24**	0.1**	2.20	0.93
C18:3 (%)	0.43**	0.15**	0.31	0.89
Oil (%)	1.65**	3.52**	1.94	0.84
Protein of defatted meal (%)	1.59**	8.01**	2.31	0.81
Thousand kernel weight (g)	0.20**	0.23**	0.26	0.85

** Denotes significance at *P* = 0.01

^a^Original values (ratio) × 100

**Table 3:** Descriptive statistic of the parents and the SODH population (*n* = 226)

Trait	Parents		Double haploid population				
	Sansibar	Oase	(*n* = 226)				
	Mean		Min	Max	Mean	*F* value	LSD
*Phytosterol* $\big( {{\text{mg }}100 {\text{g}}^{ - 1}{\text{seed}} } \big)$							
Brassicasterol	50.4	46.4	32.7	59.5	48.8	6.3**	4.6
Campesterol	157.2	114.9	87.8	192.7	136.5	13.6**	13.9
Sitosterol	226.7	167.5	154.9	251.6	193.4	6.2**	20.0
Avenasterol	23.8	19.7	9.8	52.3	25.4	6.5**	8.2
Total sterol	461.7	352.4	311.2	486.9	401.9	8.3**	34.6
24-methyl sterol	207.7	161.3	130.2	214.0	185.3	11.3**	15.7
24-ethyl sterol	250.4	187.2	170.1	252.7	218.8	7.7**	21.8
Campesterol:sitosterol^a^	69.4	68.7	47.3	99.7	71.0	13.9**	7.3
24-methyl:24-ethyl sterol^a^	82.9	86.2	62.3	108.2	85.3	12.1**	6.6
*Other traits*							
C16:0 (%)	5.0	4.6	3.8	5.6	4.8	9.6**	0.3
C18:1 (%)	58.8	63.1	57.3	65.4	61.6	11.5**	1.4
C18:2 (%)	21.0	18.7	17.1	24.1	19.9	14.1**	0.9
C18:3 (%)	9.8	9.0	7.5	11.8	9.6	9.3**	0.6
Oil (%)	43.7	46.3	41.2	48.6	45.4	6.1**	1.6
Protein of defatted meal (%)	29.3	32.9	27.3	35.2	30.5	5.1**	1.7
Thousand kernel weight (g)	5.5	5.6	4.4	7.8	5.8	5.8**	0.6

*LSD 5* *%* least significant difference at the level of 5 %

** Denotes significance at *P* = 0.01

^a^Original values (ratio) × 100

Highly significant correlations (*P* = 0.01) were observed between total phytosterol and the four individual sterols (Table 4). All nine phytosterol traits were positively correlated to C16:0 while brassicasterol in particular was correlated to all the major fatty acids. Oil was positively correlated with total phytosterol and oleic acid and negatively correlated with linoleic and linolenic acids. Except for brassicasterol, no significant correlation was observed between phytosterols and protein content of the defatted meal.

**Table 4:** Spearman’s rank correlation of traits in the SODH population (*n* = 226)

	Brassicasterol	Campesterol	Sitosterol	Avenasterol	Total phytosterol	24-Methyl sterol	24-Ethyl sterol	Campesterol:sitosterol	24-Methyl:24-ethyl sterol	C16:0	C18:1	C18:2	C18:3	Oil	Protein of defatted meal
Campesterol	0.03														
Sitosterol	0.15*	0.32**													
Avenasterol	0.04	0.78**	0.36**												
Total sterol	0.20**	0.84**	0.74**	0.80**											
24-methyl sterol	0.24**	0.98**	0.34**	0.77**	0.86**										
24-ethyl sterol	0.14*	0.53**	0.95**	0.65**	0.88**	0.54**									
Campesterol:sitosterol	−0.08	0.77**	−0.35**	0.53**	0.34**	0.73**	−0.11								
24-methyl:24-ethyl sterol	0.12	0.49**	−0.61**	0.15*	0.01	0.50**	−0.45**	0.89**							
C16:0	0.29**	0.31**	0.18**	0.26**	0.33**	0.36**	0.24**	0.18**	0.15*						
C18:1	−0.43**	−0.06	−0.09	0.04	−0.11	−0.15*	−0.06	−0.01	−0.12	−0.53**					
C18:2	0.27**	0.02	0.02	−0.09	0.03	0.08	−0.01	0.02	0.11	0.31**	−0.83**				
C18:3	0.37**	0.10	0.01	0.09	0.12	0.18**	0.04	0.08	0.13*	0.29**	−0.68**	0.34**			
Oil	−0.10	0.20**	0.16*	0.30**	0.24**	0.17**	0.23**	0.09**	−0.06	−0.02	0.48**	−0.51**	−0.23**		
Protein of defatted meal	−0.27**	0.06	−0.01	−0.05	−0.01	0.00	−0.02	0.06	0.03	−0.18**	−0.09	0.06	0.16*	−0.43**	
Seed weight	0.12	−0.08	−0.19**	−0.13	−0.15*	−0.05	−0.20**	0.05**	0.15*	−0.07	−0.11	0.14*	0.06	−0.38**	0.16*

* and ** Denotes significance at *P* < 0.05 and 0.01

### QTL mapping

**Fig. 2:**
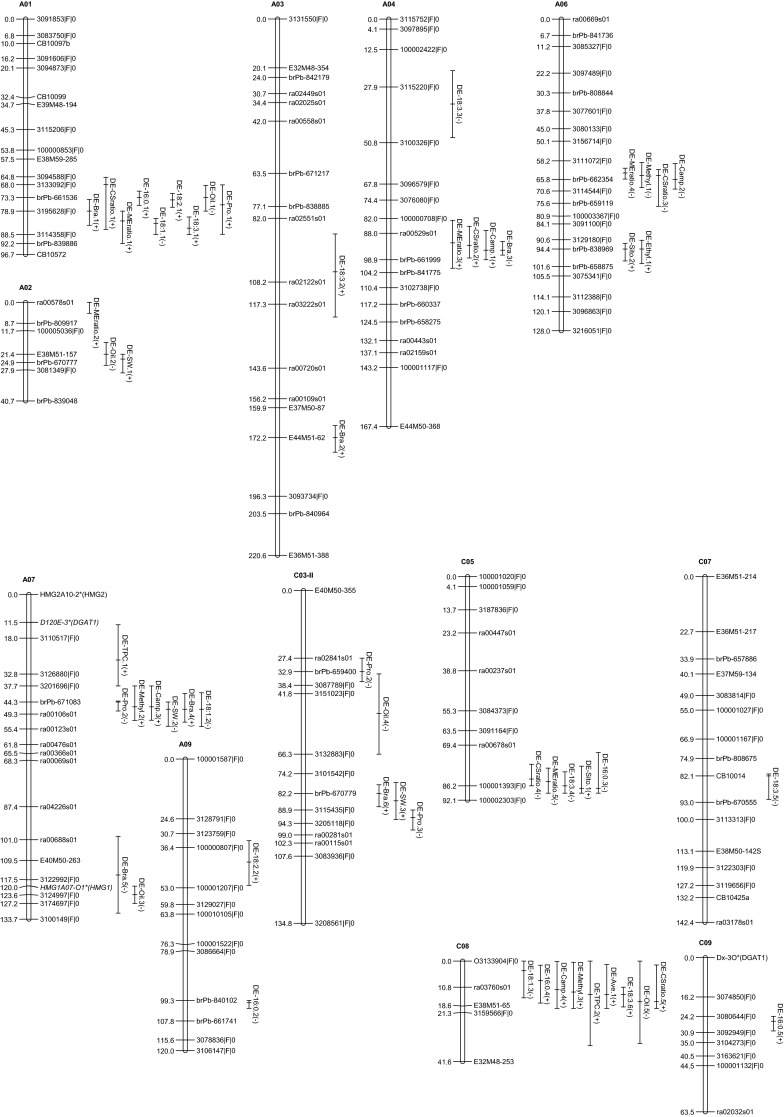
QTL associated with phytosterol traits, fatty acids, oil content, protein of defatted meal, and seed weight in SODH population. *Asterisk* on marker name indicates candidate gene-based marker. Italic font of marker name indicates placed marker. *Plus* and *minus* indicate that the trait value is increased by the allele Sansibar and Oase, respectively

Multiple interval mapping identified between one and six QTL for nine phytosterol traits, between two and six QTL for four fatty acids, five QTL for oil content, four QTL for protein content of defatted meal and three QTL for seed weight (Table 5). These QTL were distributed on 13 linkage groups as shown in Fig. 2. Colocalizations of QTL for different traits were more frequently observed than individual isolated QTL.

**Table 5:** QTL detected for phytosterol contents $\big( {{\text{mg }}100 {\text{g}}^{ - 1}{\text{seed}} } \big)$, fatty acid composition (%), oil content (%), protein content of defatted meal (%), and seed weight (g) in SODH population

Trait	QTL name	LG	Peak (cM)	CI^a^ (cM)	LOD	Additive effect^b^	*R* ^2^	Total *R* ^2^
Brassicasterol	DE-Bra.1	A01	79	74–85	5.3	0.92	4.5	62.6
	DE-Bra.2	A03	172	167–178	6.8	1.03	5.1	
	DE-Bra.3	A04	95	91–97	31.7	−2.61	38.3	
	DE-Bra.4	A07	47	41–53	5.4	0.94	4.7	
	DE-Bra.5	A07	116	100–131	3.3	−0.77	2.6	
	DE-Bra.6	C03-II	82	79–88	8.1	1.13	7.5	
Campesterol	DE-Camp.1	A04	95	87–99	6.5	5.83	11.7	37.8
	DE-Camp.2	A06	66	59–70	8.6	−6.59	13.8	
	DE-Camp.3	A07	46	38–52	3.9	4.56	5.1	
	DE-Camp.4	C08	12	0–20	4.7	4.74	7.3	
Sitosterol	DE-Sito.1	C05	87	78–89	6.0	6.15	11.3	23.2
	DE-Sito.2	A06	94	92–99	6.5	6.06	12.0	
Avenasterol	DE-Ave.1	C08	14	1–20	5.9	2.59	11.8	11.8
Total phytosterol	DE-TPC.1	A07	27	13–38	3.5	10.49	8.2	14.2
	DE-TPC.2	C08	14	0–35	3.1	8.79	6.1	
24-methyl sterol	DE-Methyl.1	A06	64	59–69	9.9	−7.73	15.9	31.1
	DE-Methyl.2	A07	46	38–52	4.7	5.40	6.9	
	DE-Methyl.3	C08	13	1–20	5.2	5.44	8.3	
24-ethyl sterol	DE-Ethyl.1	A06	94	91–100	3.6	5.92	7.2	7.2
Campesterol:sitosterol	DE-CSratio.1	A01	68	65–86	3.2	0.01	2.2	71.5
	DE-CSratio.2	A04	93	85–98	12.8	0.03	14.7	
	DE-CSratio.3	A06	64	62–77	30.4	−0.05	33.3	
	DE-CSratio.4	C05	83	77–86	16.5	−0.04	16.4	
	DE-CSratio.5	C08	17	2–20	6.1	0.02	4.9	
24-methyl: 24-ethyl sterol	DE-MEratio.1	A01	83	79–92	5.2	0.02	3.6	70.3
	DE-MEratio.2	A02	25	0–5	4.9	0.02	4.5	
	DE-MEratio.3	A04	92	83–102	4.2	0.02	5.6	
	DE-MEratio.4	A06	63	61–66	32.3	−0.05	38.7	
	DE-MEratio.5	C05	84	79–89	16.5	−0.03	17.8	
C16:0	DE-16:0.1	A01	73	71–77	6.2	0.08	4.8	59.0
	DE-16:0.2	A09	100	99–103	21.7	−0.30	28.8	
	DE-16:0.3	C05	87	73–89	3.9	−0.07	4.0	
	DE-16:0.4	C08	8	2–17	12.2	0.12	10.4	
	DE-16:0.5	C09	26	24–30	8.1	0.10	10.9	
C18:1	DE-18:1.1	A01	84	82–89	16.5	−0.87	26.3	43.6
	DE-18:1.2	A07	47	41–54	3.7	−0.39	5.6	
	DE-18:1.3	C08	4	0–15	8.1	−0.60	11.7	
C18:2	DE-18:2.1	A01	74	72–77	11.2	0.53	18.8	30.6
	DE-18:2.2	A09	42	34–52	5.0	0.43	11.8	
C18:3	DE-18:3.1	A01	86	81–89	20.0	0.36	27.3	57.0
	DE-18:3.2	A03	104	88–123	3.9	0.15	4.8	
	DE-18:3.3	A04	35	21–49	3.2	−0.16	7.4	
	DE-18:3.4	C05	86	80–89	4.7	−0.17	3.5	
	DE-18:3.5	C07	82	81–92	5.0	−0.16	5.3	
	DE-18:3.6	C08	14	11–19	8.2	0.21	8.7	
Oil content	DE-Oil.1	A01	73	68–79	3	−0.31	4.3	27.5
	DE-Oil.2	A02	21	16.5–26	5	−0.39	6.3	
	DE-Oil.3	A07	124	120–127	5	−0.44	6.7	
	DE-Oil.4	C03-II	50	34–66	3	−0.37	4.7	
	DE-Oil.5	C08	17	0–34	3	−0.30	5.5	
Protein of defatted meal	DE-Pro.1	A01	76	68–88	5.2	0.40	8.7	38.1
	DE-Pro.2	A07	44	44–48	6.7	−0.44	8.5	
	DE-Pro.3	C03-II	33	27–37	4.6	−0.36	8.0	
	DE-Pro.4	C03-II	92	89–97	6.9	−0.46	12.9	
Seed weight	DE-SW.1	A02	23	21–29	5.5	0.15	10.3	27.1
	DE-SW.2	A07	47	44–54	5.5	−0.16	10.7	
	DE-SW.3	C03-II	85	78–93	3.0	0.12	6.1	

*LG* linkage group

^a^ *CI* 1-LOD confidence interval

^b^Additive effect is the substitution effect of one Oase allele by one Sansibar allele

#### Phytosterols

QTL for phytosterols content were distributed on nine linkage groups: A01, A02, A03, A04, A06, A07, C03-II, C05, and C08 (Table 5; Fig. 1). Major QTL (*R*^2^ ≥ 25 %) were identified for brassicasterol on linkage group A04 (*DE*-*Bra.3*), for campesterol to sitosterol ratio and 24-methyl to 24-ethyl sterol ratio on linkage group A06 (*DE*-*CSratio.3 and DE*-*MEratio.4*). The major QTL *DE*-*Bra.3* on A04 was at the same position as the QTL for campesterol, and overlapped with QTL for the campesterol to sitosterol ratio and 24-methyl to 24-ethyl sterol ratio; the additive effect of *DE*-*Bra.3* was negative as opposed to the other three QTL. On the contrary, minor QTL for brassicasterol on A01 (*DE*-*Bra.*1) and A07 (*DE*-*Bra.4*) overlapped with QTL for phytosterol related traits with the same direction of additive effect. The major QTL *DE*-*CSratio.3* and *DE*-*MEratio* on A06 overlapped with QTL for campesterol and 24-methyl sterol; these four QTL showed negative effects, indicating that the alleles increasing the trait values were derived from Oase. For total phytosterol content, two QTL with positive additive effects were detected on A07 and C08; the QTL *DE*-*TPC.1* on A07 was located at the top of the linkage group, close to a genomic region with many QTL (Table 5; Fig. 2). The QTL *DE*-*TPC.2* on C08 overlapped with QTL for different phytosterols as well as for fatty acids and oil content.

#### Fatty acids, oil content, and protein content of the defatted meal

Between two and six QTL were identified for fatty acids, five QTL for oil content, and four QTL for protein content of the defatted meal (Table 5; Fig. 2). Major QTL were detected for C16:0 on A09 (DE-16:0.2) and for oleic acid and linolenic acid on A01 (*DE*-*18:1.1* and *DE*-*18:3.1*). Only minor QTL were detected for oil content and for protein content of the defatted meal which individually explained between 4.3 and 6.7 % and between 8.0 and 12.9 % of the phenotypic variance, respectively. There were two QTL hotspot regions worth mentioning. The first one consists of QTL *DE*-*16:0.1*, *DE*-*18:1.1*, *DE*-*18:2.1*, *DE*-*Oil.1,* and *DE*-*Pro.1* on A01. The direction of the additive effects suggests that the Oase allele led to a decrease of C16:0, C18:2, C18:3, and protein content of the defatted meal, and to an increase in C18:1 and oil content. The second QTL hotspot region consists of QTL *DE*-*16:0.4*, *DE*-*18:1.3*, *DE18:3.6*, and *DE*-*Oil.5* on C08; the directions of the additive effects were identical to the QTL at the first QTL hotspot region. All of the five QTL for oil content had negative additive effects, indicating that the alleles increasing the oil content were derived from Oase. Interestingly, the largest QTL *DE*-*Oil.3* on A07 was located within the confidence interval of QTL *DE*-*Bra.5* for brassicasterol with the same direction of the additive effect.

#### Seed weight

The three QTL detected for seed weight were found on linkage groups A02, A07, and C03-II (Table 5; Fig. 2). Individual QTL explained between 6.1 and 10.7 % of the phenotypic variance, which collectively accounted for 27.1 % of the total phenotypic variance. Additive effects were positive for QTL located on A02 and C03-II and negative for QTL located on A07. The QTL *DE*-*SW.1* on A02 overlapped with QTL *DE.Oil.2* with opposite effects, indicating that the Oase allele was increasing oil content and decreasing seed weight. The QTL *DE*-*SW.2* on A07 overlapped with QTL *DE*-*Bra.4,DE*-*18:1.2,DE*-*Methyl.2*, *DE*-*Camp.3*, and *DE*-*Pro.2*; the direction of the additive effects indicate that the Oase allele led to an increase in seed weight, oleic acid, and protein content of the defatted meal and to a decrease in phytosterols. The QTL *DE*-*SW.3* on C03-II overlapped with QTL *DE*-*Pro.4* and *DE*-*Bra.6*; the directions of the additive effects indicate that the Oase allele reduced seed weight and Brassicasterol content but increased protein content of the defatted meal.

### Identification of possible candidate genes for major QTL

Sequences of markers associated with the major QTL and the predicted genes were searched in genome sequences of both *B. rapa* Chiifu (Wang et al. 2011) and *B. napus* Darmor-*bzh* (Chalhoub et al. 2014). The congruency of marker orders was better with *B. rapa* Chiifu genome sequence assembly while some markers and most of the predicted genes were found matching on random, non-anchored scaffolds in *B. napus* Darmor-bzh genome assembly. Therefore, the *B. rapa* Chiifu genome sequence assembly was used as a reference for the investigation of the underlying candidate genes.

The alignments of the major QTL on A01, A04, A06, and A09 with *B. rapa* are provided in Supplementary Figs. 3–6. Within the genomic region of 64.8–92.2 cM on A01, major QTL for C18:1 and C18:3 were found colocalized with *FAD2* (not annotated in *B. rapa*) while the minor QTL for C16:0, C18:2, and oil content were colocalized with *LPAAT* (Bra037553). The major QTL for brassicasterol on A04 colocalized with two orthologs of *CYP710A2* which are annotated as *CYP710A1* in *B. rapa* (Bra021916 and Bra021917). On A06, major QTL for campesterol to sitoterol ratio and 24-methyl to 24-ethyl sterol ratio colocalized with *SMT2.* On A09, the major QTL for C16:0 colocalized with the homolog of *FATB* (Bra031631).

## Discussion

### Molecular markers and linkage map

The two parental lines, Sansibar and Oase, revealed a narrow genetic background based on their low level of polymorphisms for most marker types. The numbers of polymorphic markers were greatly increased with array-based high-throughput DArT and silico-DArT markers which at the same time were also sequence informative. As a 10-cM interval between marker loci is commonly used for QTL analysis, the SODH map can be considered suitable for performing QTL analysis. High level of segregation distortions observed in this study have also been reported in other *B. napus* maps (Kaur et al. 2009; Zhang et al. 2011; Delourme et al. 2013; Raman et al. 2013). Such phenomenon appears to be common in maps of microspore-derived DH populations, which may be due to the differential responsiveness between the two parental lines to microspore culture during in vitro androgenesis and plantlet regeneration (for a review see Ferrie and Möllers 2011). The SODH map has a higher number of markers mapped on the A genome than on the C genome, similar to a few reported studies (Bancroft et al. 2011; Delourme et al. 2013; Raman et al. 2013).

### Phenotypic analysis

Results from the phenotypic analysis revealed a relatively large and significant phenotypic variation for all the traits. Total phytosterol content which ranged from 311.2 to 486.9 mg 100 g^−1^ seed was comparable to the range from 356.6 to 480.0 mg 100 g^−1^ seed reported in 27 modern rapeseed cultivars (Amar et al. 2009) and higher than the range from 257 to 410 mg 100 g^−1^ seed reported in a DH population segregating for erucic acid content (Amar et al. 2008b). By taking oil content into consideration, the theoretical phytosterol content in oil ranged from 718 to 1123 mg 100 g^−1^ oil in the SODH population, which was lower than the range from 766 to 1402 mg 100 g^−1^ oil in 12 different spring canola varieties (Abidi et al. 1999) but higher than the range from 464 to 807 mg 100 g^−1^ oil in nine canola lines (Vlahakis and Hazebroek 2000) and the range from 448 to 928 mg 100 g^−1^ oil in three different DH populations of winter oilseed rape (Amar et al. 2008b). The high total phytosterol content found in the SODH population may be attributed to the low erucic acid content of the seed oil as a negative correlation between the two traits has been reported by Amar et al. (2008b). Among the individual phytosterols, sitosterol was the most prominent sterol, followed by campesterol, brassicasterol, and avenasterol, which is in accord with the relative contents reported from literatures (Vlahakis and Hazebroek 2000; Verleyen et al. 2002; Amar et al. 2008a, b, 2009). The transgressive segregation for individual and total phytosterol content of the DH population can be explained by the fact that both parents contributed positive alleles. The range of seed oil content from 41.2 to 48.6 % was within the range of commercial cultivars which usually contain about 40–50 % of oil. Significant genotypic variation and high heritability observed in all traits suggest that SODH population is suitable for QTL analysis. From a nutritional point of view, negative correlations between oil content and polyunsaturated fatty acids such as linoleic and linolenic acids and positive correlations between oil content and both oleic acid and total phytosterol content are desirable as reduced levels of polyunsaturated fatty acids and increased level of oleic acid will increase oxidative stability of oil while phytosterols can lower LDL cholesterols.

### QTL mapping

Results from multiple interval mapping (MIM) indicate that additive effects were the main factors contributing to variation in all traits as no significant epistatic interaction was detected in any case. For total phytosterol content, the present study identified only two minor QTL located on A07 and C08 while Amar et al. (2008a) detected two major QTL on A08 and C03 and a minor QTL on C08. The disappearance of two major QTL in the present study corroborate the findings of Amar et al. (2008a) who found that the two major QTL for total phytosterol content were most likely due to pleiotropic effects exerted by the erucic acid genes. As a matter of fact, the present study did not detect any QTL on A08 and C03 for all the nine phytosterol traits except for the one minor QTL for brassicasterol identified on C03-II (*DE*-*Bra.6*). By disregarding the QTL on A08 and C03 from the study of Amar et al. (2008a, b), the number of QTL was almost the same as detected in the present study except for avenasterol in which four additional QTL were detected in the study of Amar et al. (2008a). Similarly, the present study shows that more QTL were detected for individual phytosterol content than for total phytosterol content. Of the eight linkage groups that harbored QTL for phytosterols in this study, only two linkage groups (A02 and A07) were not found to have QTL in the study of Amar et al. (2008a).

#### Major QTL and possible underlying candidate genes

Of the 16 traits analyzed, QTL with major effects were found for C18:1 and C18:3 on A01, brassicasterol on A04, campesterol to sitosterol ratio and 24-methyl to 24-ethyl sterol ratio on A06, and C16:0 on A09. A good colinearity observed between the genetic and the *B. rapa* physical map positions enabled the investigation of the possible underlying candidate genes.

On A01, major QTL for C18:1 and C18:3 colocalized with *FAD2* which encodes the enzyme endoplasmic Δ-12 oleate desaturase that desaturates C18:1 to C18:2. In *B. napus*, four loci located on A01, A05, C01, and C05 have previously been reported for *FAD2* (Schierholt et al. 2000). However, in the reference genome of *B. napus*, homologs of *FAD2* were only found on A05, C05, and scaffold chromosome. Because there were many cases like this, where the position of the candidate gene could not be determined, the reference genome of *B. rapa* was used for the investigation of candidate genes. About 2–3 cM above the major QTL for C18:1 and C18:3, there were minor QTL for C16:0, C18:2, and oil content which were found colocalized with the *LPAAT* gene, which encodes the second enzyme of the Kennedy pathway that acylates the *sn*-*2* hydroxyl group of lysophosphatidic acid to form phosphatidic acid. In *Arabidopsis*, expression of the oilseed rape microsomal LPAAT isozyme has shown enhancement of seed oil content and seed mass (Maisonneuve et al. 2010). Moreover, studies have shown that LPAAT is a substrate-specific acyltransferase and appears to discriminate against saturated acyl CoA in most oilseeds (Norton and Harris 1983; Sun et al. 1988; Lassner et al. 1995), which somehow explains the opposite direction of additive effects observed between QTL for oil content (*DE*-*Oil.1*) and QTL for palmitic acid and oleic acid (*DE*-*16:0.1* and *DE*-*18:2.1*). In addition, the negative correlation between palmitic acid and oleic acid (*r*_s_ = −0.53**) in SODH population may suggest an enhanced flux in *de novo* fatty acid synthesis pathway. As reported by Möllers and Schierholt (2002), an enhanced C16/C18-fatty acid ratio of the seed oil may indicate an improved seed oil synthesis by a top-down control mechanism. In maize, simultaneous enhancement of oil and oleic acid contents in seed are linked to a gene encoding diacylglycerol acyltransferase (DGAT) that catalyzes the final committed step in the Kennedy pathway leading to triacylglycerol production (Zheng et al. 2008). Similarly, expression of DGAT in wild-type *Arabidopsis thaliana* and the high linoleic acid *fad3fae1* mutant have both shown a striking increase in seed oleic acid content (Zhang et al. 2013). One explanation for the influence of DGAT on fatty acid composition, or more specifically the increased oleic acid content, is that the enhanced triacylglycerol synthesis mediated by DGAT limits the flux through the phosphatidylcholine-based desaturation reactions (Aznar-Moreno et al. 2015). In our study, we show that the positive correlation between oil and oleic acid content could also be due to close linkage between *FAD2* and *LPAAT* (Supplementary Fig. 3). Because the nature of metabolic control is such that a single gene (or enzyme) rarely leads to a huge effect for a complex trait like oil content, recognizing the pleiotropic effect and close linkage of the involved genes may facilitate gene stacking for improving seed oil content as well as the quality traits. Therefore, it would also be of interest to identify the functional polymorphisms of *LPAAT* and *FAD2* between Sansibar and Oase as well as to investigate the allelic diversity using a broader germplasm to develop functional marker for marker-assisted selection.

The major QTL *DE*-*Bra.3* on A04 collocated with two orthologs of *CYP710A2*, annotated as CYP710A1 in *B. rapa.* The CYP710A genes have been known to encode cytochrome P450 enzyme that catalyzes the C-22 desaturation reaction, converting both 24-epi-campesterol and sitosterol to brassicasterol and stigmasterol, respectively (Morikawa et al. 2006). In Arabidopsis, three C-22 sterol desaturases encoded by *CYP710A1*, *CYP710A2*, and *CYP710A4* (Morikawa et al. 2006; Arnqvist et al. 2008) are able to catalyze the synthesis of stigmasterol while only one C22-desaturase encoded by *CYP710A2* is able to produce brassicasterol (Morikawa et al. 2006). The fact that QTL *DE*-*Bra.3* overlapped with three other QTL (*DE*-*Camp.1, DE*-*CSratio.2 and DE*-*MEratio.3*) with opposite effects also suggests that *CYP710A1* gene exerts a pleiotropic effect on the composition of phytosterols. Given that brassicasterol is synthesized via two enzymatic steps from 24-methylene cholesterol, and campesterol is synthesized directly from 24-methylene cholesterol, a trade-off between campesterol and brassicasterol is usual in the case of parallel biosynthetic pathways (Fig. 1). Overlapping QTL between brassicasterol and campesterol on A04 as well as the opposite additive effects were similarly observed in the study of Amar et al. (2008a), indicating that they may be the same loci in both populations.

On A06, two major QTL for campesterol to sitosterol ratio and 24-methyl to 24-ethyl sterol ratio colocalized with two other minor QTL for campesterol and 24-methyl sterol (Table 5; Fig. 2). About 30 cM below this genomic region, there was a colocation of two minor QTL for sitosterol and 24-ethyl sterol with positive additive effects as opposed to the upper genomic region. Alignment between the genomic region and physical map of *B. rapa* revealed that the SMT2 gene was within the genomic region of major QTL (Supplementary Fig. 5). The *SMT2* gene encodes the enzyme sterol methyltransferase 2 which regulates the ratio between campesterol and sitosterol or between 24-methyl sterol and 24-ethyl sterol. Campesterol to sitosterol ratio is of interest because it is important in plant growth and development (Schaeffer et al. 2001) and in humans, it determines the efficacy of cholesterol lowering ability (Miettinen 2001). The ability of plants to synthesize sterols with branched ethyl groups (as in sitosterol and stigmasterol) has also been proposed to be part of an evolutionary adaptation process to cope with wider temperature fluctuations, and to maintain the essential membrane associated metabolic processes, as compared to animals (Dufourc 2008). Considering the fact that no unfavorable pleiotropic effect or close linkage with other quality traits were observed, independent effect of this major QTL or *SMT2* on A06 could be of interest for modifying phytosterol composition.

On A09, the major QTL *DE*-*16:0.2* for palmitic acid was found colocalized with the *FATB* gene (Table 5; Supplementary Fig. 6), which encodes the enzyme that hydrolyzes the thioester bond of C16:0-ACP and releases C16:0 from acyl-ACP (Bonaventure et al. 2003). Acyl-ACP thioesterases are known to be responsible for regulating the chain termination during *de novo* fatty acid synthesis and in channeling carbon flux between the plastid and cytosol in plants. The *FATB* gene belongs to one of the two isoforms of acyl-ACP thioesterase which primarily hydrolyzes C8–C16-saturated acyl-ACPs (Jones et al. 1995). Given that no other QTL were found overlapping with *DE*-*16:0.2*, this further supports the hypothesis that *FATB* is the underlying candidate gene. Similar assumption can also be made for QTL *DE*-*16:0.5* on C09 but the homologs of *FATB* in *B. napus* C subgenome were located on C05 and C08.

#### Minor QTL

In contrast to the large effects of QTL identified for phytosterols and fatty acids, five minor QTL distributed on five linkage groups were identified for oil content. The alleles increasing oil content were all derived from Oase, the parent with a high oil content, which explains why only slight transgressive segregation was observed in the SODH population. The SODH population is similar to the RNSL population used in the study of Delourme et al. (2006) as the parents were also chosen from the elite winter oilseed rape germplasm which differs in oil content. The study reported a total of 10 genomic regions associated with oil content which were distributed on 10 linkage groups. A comparison between the two populations showed that QTL were similarly detected on five linkage groups (A01, A07, A08, C03, and C08) but it could not be confirmed if they were the same loci in both populations as the genetic maps do not share any common markers. In this study, the QTL with the largest effect was located on A07 (*DE*-*Oil.3*) and was found colocalized with QTL for brassicasterol (*DE*-*Bra.5*) and the candidate gene-based marker of *HMG1* (HMG1A07O) at 120 cM. Given that *HMG1* gene is responsible for regulating the carbon flux into the isoprenoid pathway and both de novo fatty acid synthesis and phytosterol synthesis share the same precursor (acetyl-CoA) (Fig. 1), the collocation of both QTL with *HMG1* may be caused by a downstream effect of *HMG1* gene or alternatively, it might be due to close linkage between the causative genes and *HMG1*. Besides the *HMG1*, two other candidate genes, *HMG2* and *DGAT1*, were also mapped on A07 but were not found colocated with any QTL. Above the overlapping QTL for oil and brassicasterol on A07 lies a genomic region (38–54 cM) which harbored six QTL associated with different traits (brassicasterol, campesterol, 24-methyl sterol, oleic acid, protein of defatted meal, and seed weight). All of the six QTL showed minor effects; however, QTL for protein content of defatted meal and seed weight were the individual QTL which have the largest effect in their respective trait. Particularly for seed weight, numerous studies have consistently detected QTL on A07 in different populations with diverse genetic backgrounds (Quijada et al. 2006; Udall et al. 2006; Shi et al. 2009; Basunanda et al. 2010; Cai et al. 2012) while in the latest study, 12 candidate genes underlying 8 QTL for seed weight were identified through comparative mapping among *Arabidopsis* and *Brassica* species but no candidate genes could be inferred for the two major QTL detected on A07 (Cai et al. 2012). In conclusion, the results show that phytosterol composition and content can be improved without hampering genetic progress in improving seed oil content. Major QTL were found exclusively on the A genome and the identified candidate genes would need to be confirmed in future studies for implementing marker-assisted selection. Notably, the colocation of QTL for oil content and fatty acids with *LPAAT* and *FAD2* on A01 could either be due to independent or combined effects of the genes.

## Electronic supplementary material

Supplementary material 1 (DOCX 72 kb)

Supplementary material 2 (DOCX 38 kb)

Supplementary material 3 (DOCX 91 kb)

Supplementary material 4 (DOCX 94 kb)

Supplementary material 5 (DOCX 153 kb)

Supplementary material 6 (DOCX 119 kb)

Supplementary material 7 (DOCX 15 kb)

Supplementary material 8 (DOCX 12 kb)

Supplementary material 9 (XLSX 150 kb)

Supplementary material 10 (XLSX 368 kb)
