# Supplementary material for: Genetic variation and inheritance of phytosterol and oil content in a doubled haploid population derived from the winter oilseed rape Sansibar × Oase cross
Source: Theor Appl Genet. 2015 Oct 30;129:181–99. doi: 10.1007/s00122-015-2621-y (PMC4703628; doi:10.1007/s00122-015-2621-y)
Supplement: Supplementary file 1 — Supplementary material 1 (DOCX 72 kb) [file 122_2015_2621_MOESM1_ESM.docx]

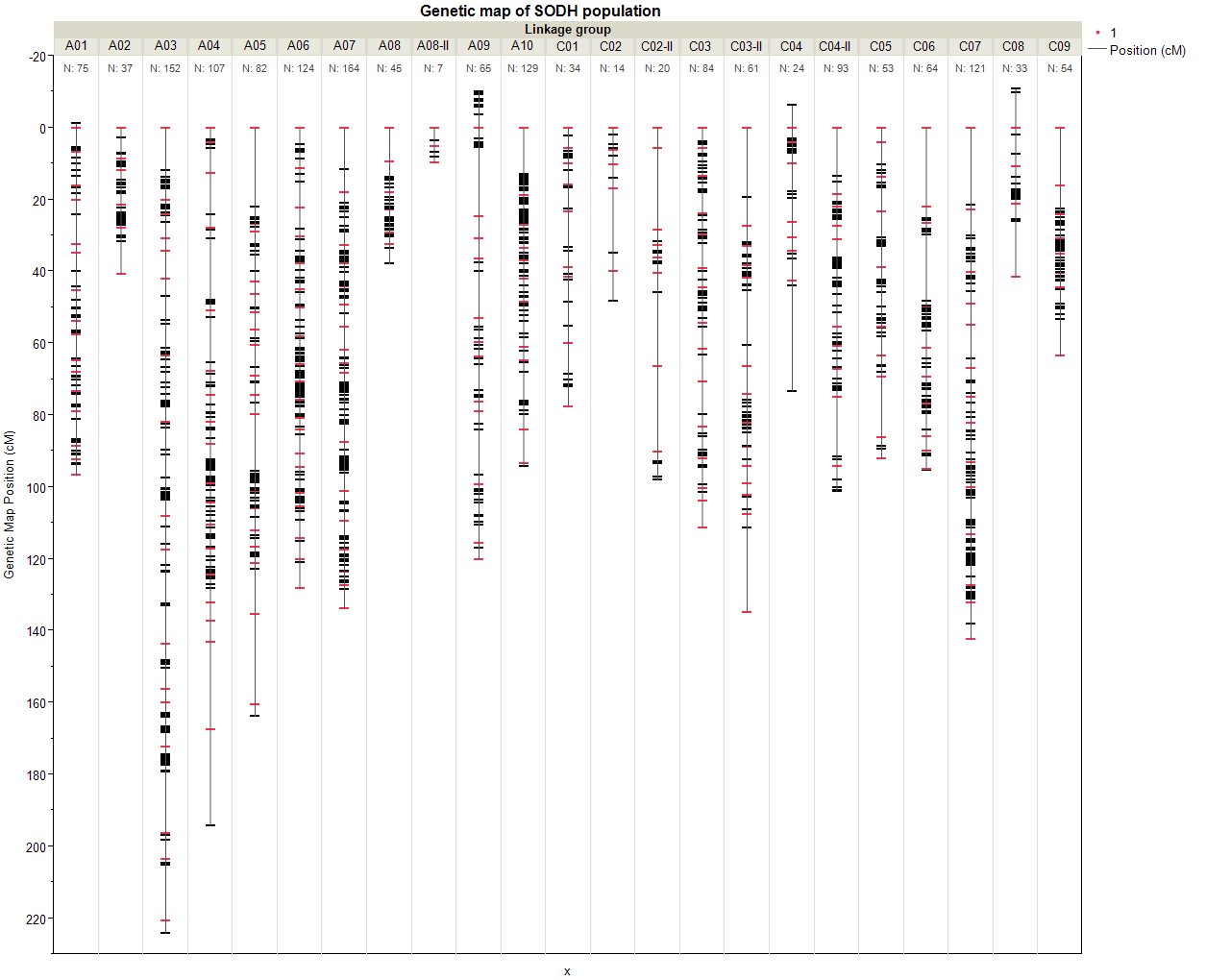


Supplementary Figure 1. The genetic map of the SODH population constructed based on a total of 1642 markers, organized in 23 linkage groups and covering a map length of 2350 cM with a mean marker interval of 2.0 cM. Framework markers used in QTL mapping are indicated in red.
