# Supplementary material for: Genetic variation and inheritance of phytosterol and oil content in a doubled haploid population derived from the winter oilseed rape Sansibar × Oase cross
Source: Theor Appl Genet. 2015 Oct 30;129:181–99. doi: 10.1007/s00122-015-2621-y (PMC4703628; doi:10.1007/s00122-015-2621-y)
Supplement: Supplementary file 2 — Supplementary material 2 (DOCX 38 kb) [file 122_2015_2621_MOESM2_ESM.docx]

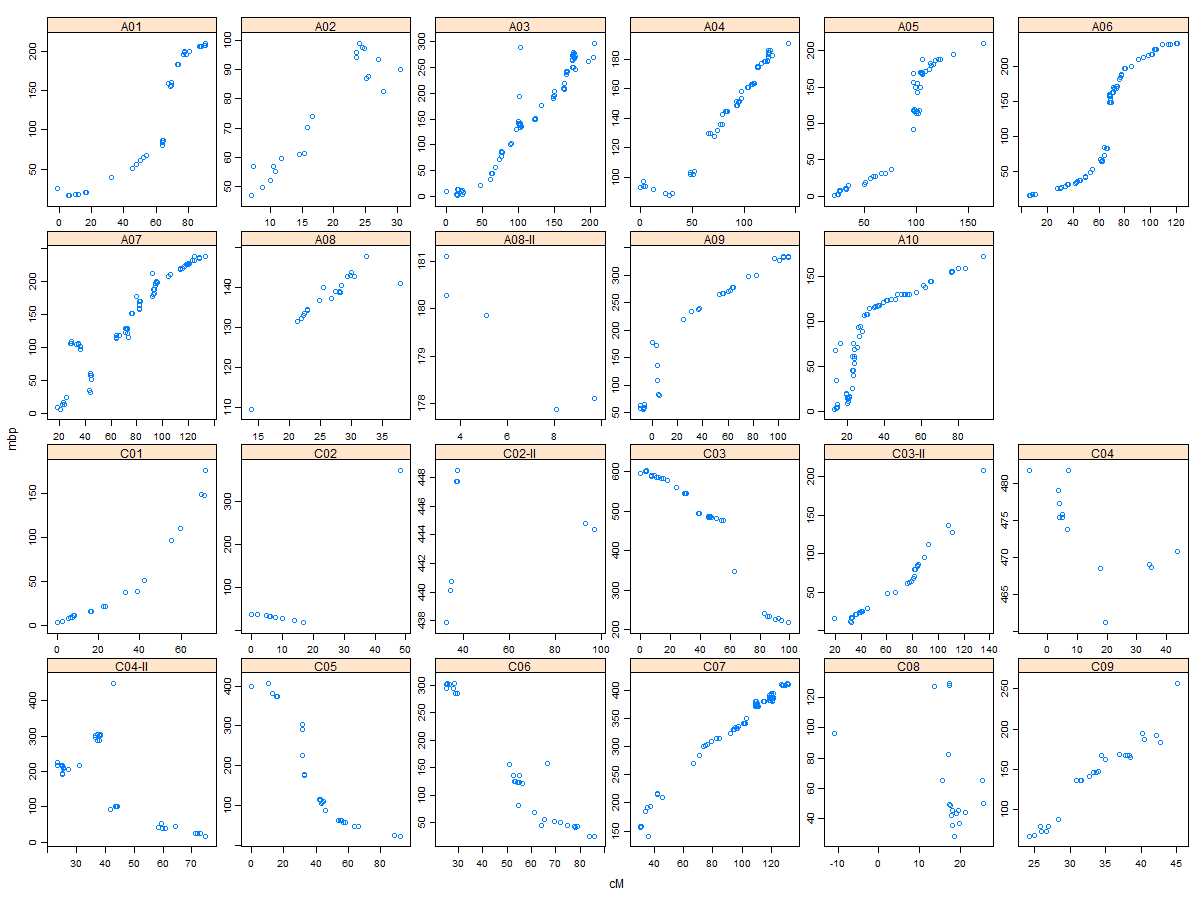


Supplementary Figure 8. Alignment of SODH map with the physical map of B. napus Darmor-bzh genome assembly
