# Supplementary material for: Genetic variation and inheritance of phytosterol and oil content in a doubled haploid population derived from the winter oilseed rape Sansibar × Oase cross
Source: Theor Appl Genet. 2015 Oct 30;129:181–99. doi: 10.1007/s00122-015-2621-y (PMC4703628; doi:10.1007/s00122-015-2621-y)
Supplement: Supplementary file 3 — Supplementary material 3 (DOCX 91 kb) [file 122_2015_2621_MOESM3_ESM.docx]

Supplementary Figure 3


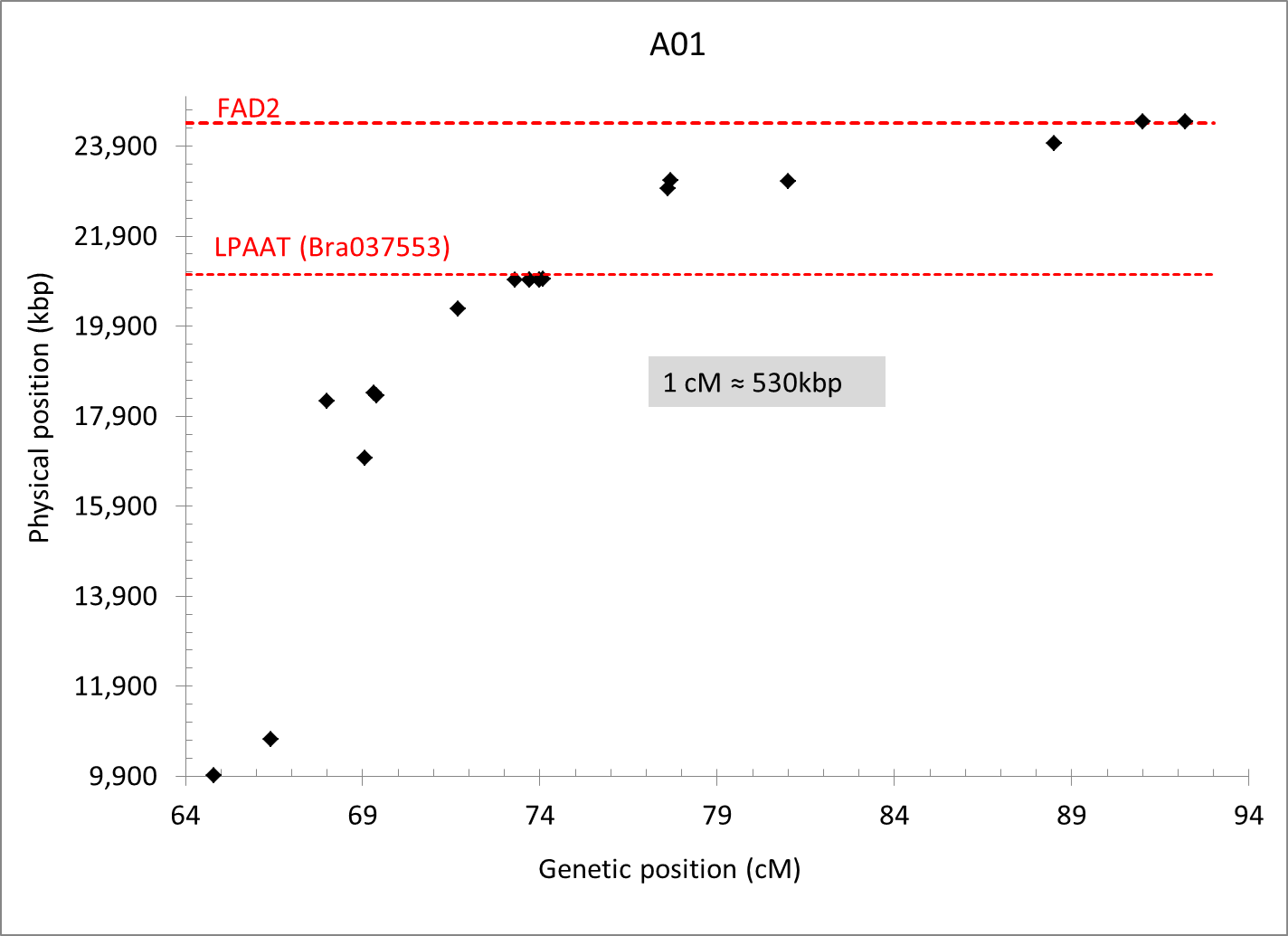


1. Alignment of genetic and physical map positions of markers within the QTL genomic region on A01. The physical position of candidate genes (*FAD2*) and (*LPAAT*) are indicated by the red dotted line


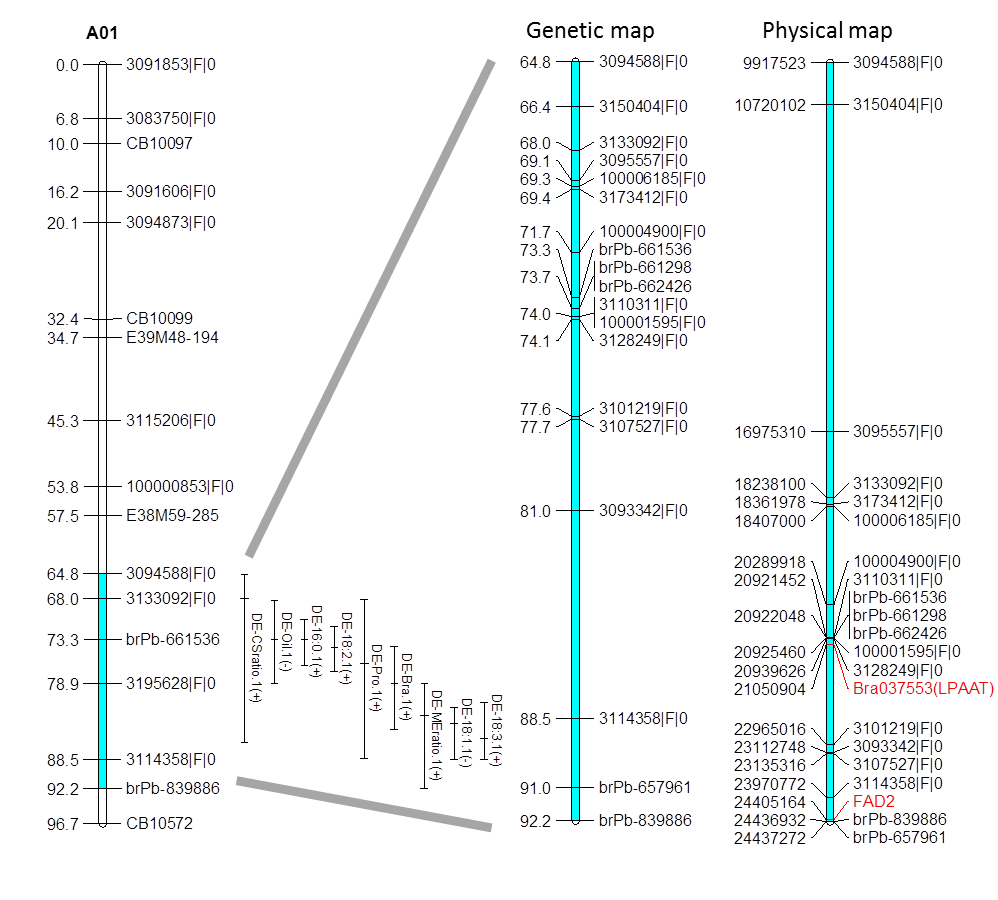


1. Genetic and physical map positions of markers within the QTL genomic region (64.8-92.2 cM) on A01. Left: QTL mapped on A01 in framework map of SODH population. Middle: Additional markers mapped within the QTL genomic region in full map of SODH population right: The corresponding physical positions of additional markers and the candidate genes (*FAD2*) and (*LPAAT*) in *B. rapa* genome
