# Supplementary material for: Genetic variation and inheritance of phytosterol and oil content in a doubled haploid population derived from the winter oilseed rape Sansibar × Oase cross
Source: Theor Appl Genet. 2015 Oct 30;129:181–99. doi: 10.1007/s00122-015-2621-y (PMC4703628; doi:10.1007/s00122-015-2621-y)
Supplement: Supplementary file 4 — Supplementary material 4 (DOCX 94 kb) [file 122_2015_2621_MOESM4_ESM.docx]

## Supplementary Figure 4


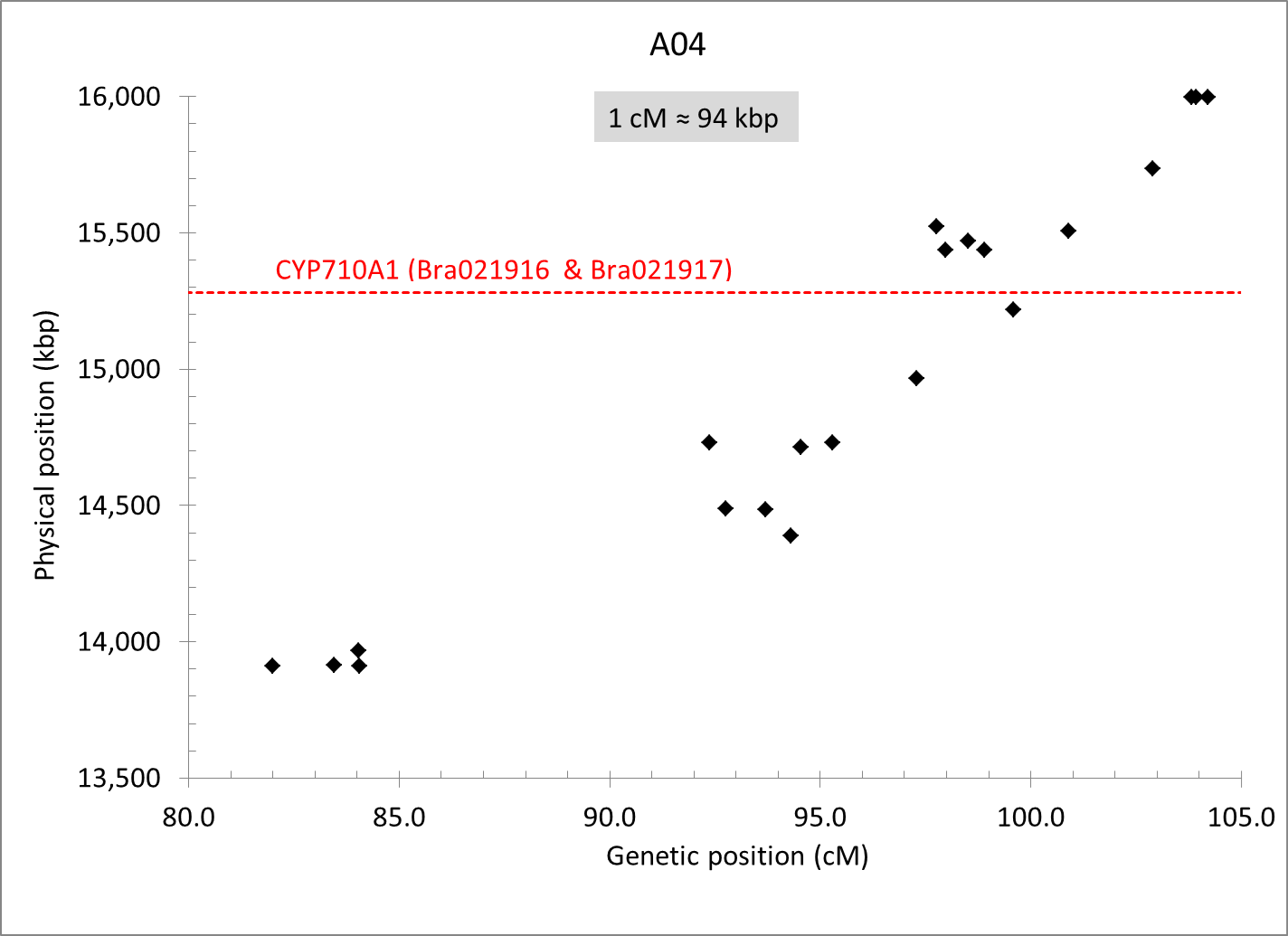


1. Alignment of genetic and physical map positions of markers within the QTL genomic region (82-104.2 cM) on A04. The physical position of candidate gene (CYP710A1) is indicated by the red dotted line.


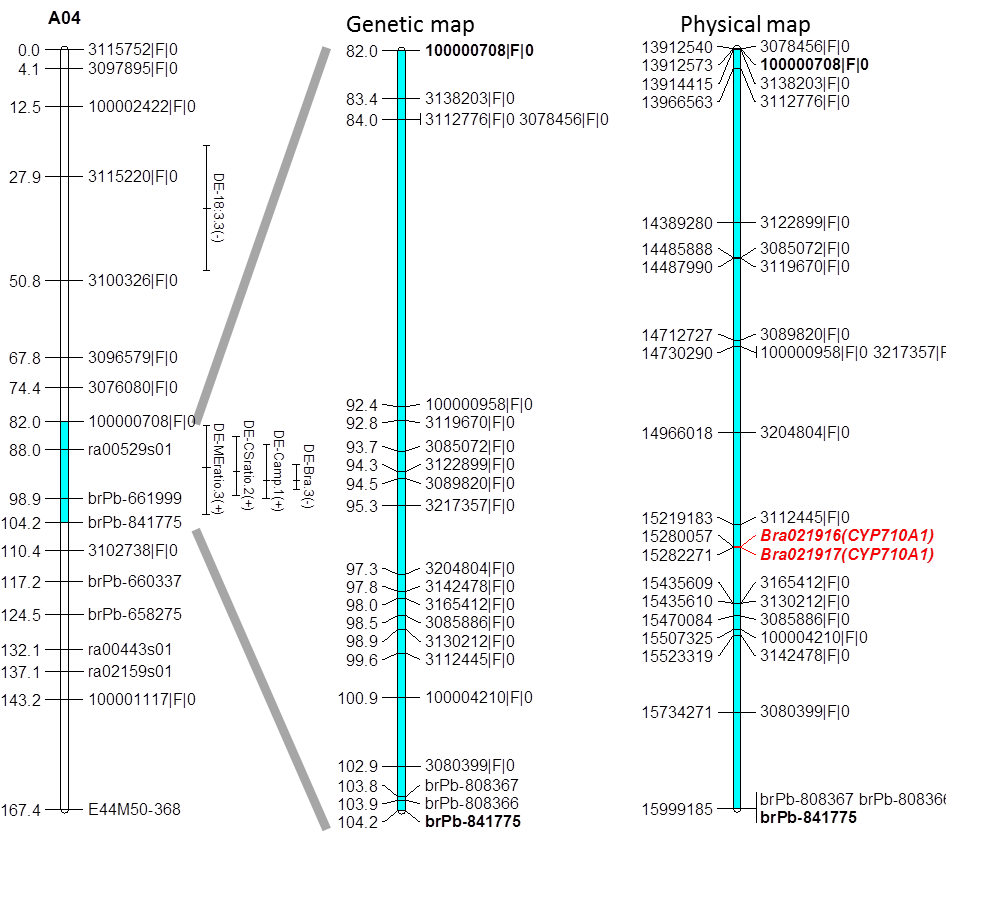


1. Genetic and physical map positions of markers within the QTL genomic region (82-104.2 cM) on A04. Left: QTL mapped on A04 in framework map of SODH population. Middle: Additional markers mapped within the QTL genomic region (82-104.2 cM) in full map of SODH population Right: The corresponding physical positions of additional markers and the candidate gene (CYP710A1) in *B. rapa* genome.
