# Supplementary material for: Genetic variation and inheritance of phytosterol and oil content in a doubled haploid population derived from the winter oilseed rape Sansibar × Oase cross
Source: Theor Appl Genet. 2015 Oct 30;129:181–99. doi: 10.1007/s00122-015-2621-y (PMC4703628; doi:10.1007/s00122-015-2621-y)
Supplement: Supplementary file 5 — Supplementary material 5 (DOCX 153 kb) [file 122_2015_2621_MOESM5_ESM.docx]

## Supplementary Figure 5


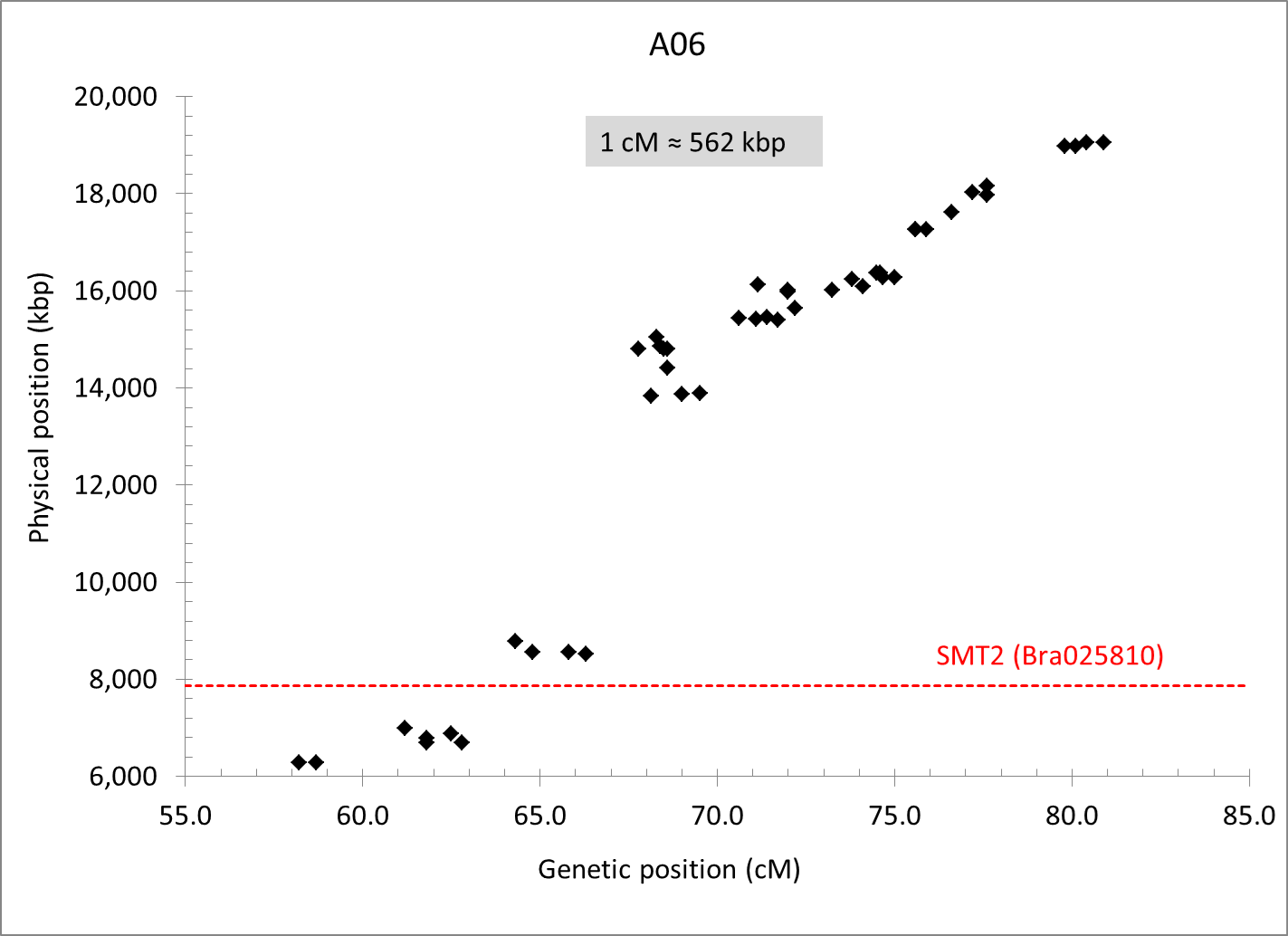


1. Alignment of genetic and physical map positions of markers within the QTL genomic region on A06. The physical position of the candidate gene (SMT2) is indicated by the red dotted line


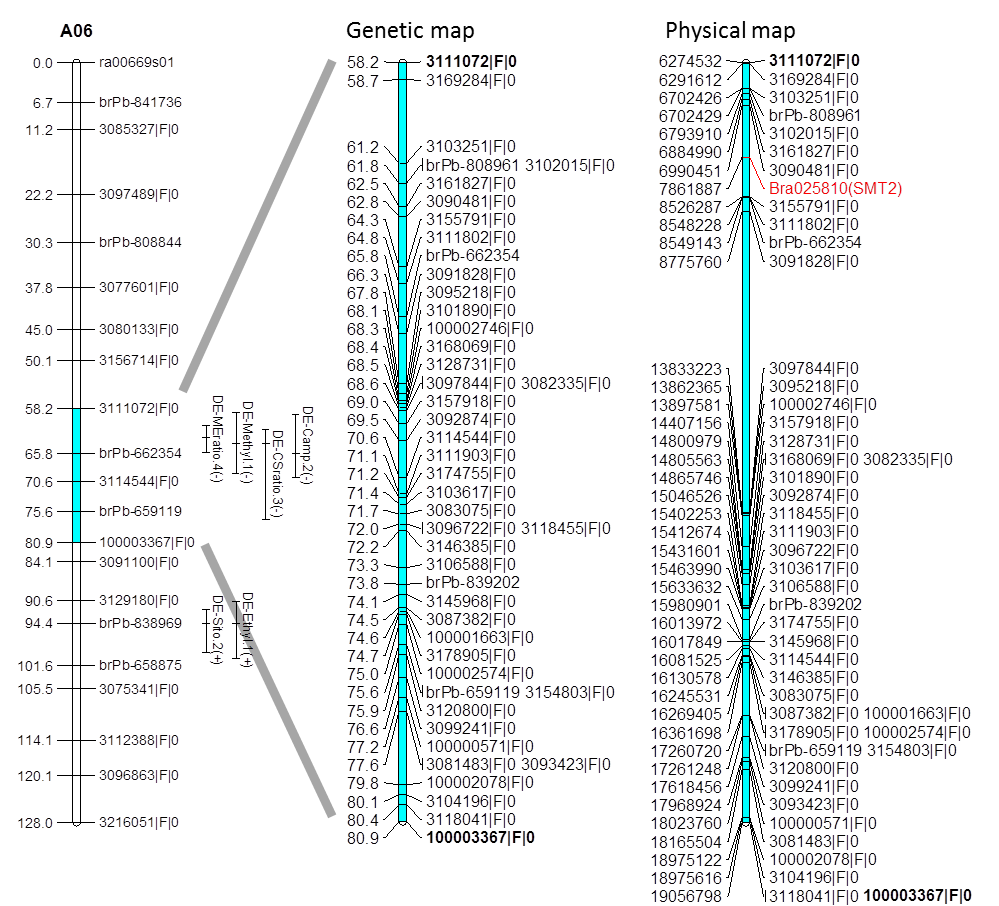


1. Genetic and physical map positions of markers within the QTL genomic region (58.2-80.9 cM) on A06. Left: QTL mapped on A04 in framework map of SODH population. Middle: Additional markers mapped within the QTL genomic region in full map of SODH population Right: The corresponding physical positions of additional markers and the candidate gene (SMT2) in B. rapa genome
