# Supplementary material for: Genetic variation and inheritance of phytosterol and oil content in a doubled haploid population derived from the winter oilseed rape Sansibar × Oase cross
Source: Theor Appl Genet. 2015 Oct 30;129:181–99. doi: 10.1007/s00122-015-2621-y (PMC4703628; doi:10.1007/s00122-015-2621-y)
Supplement: Supplementary file 7 — Supplementary material 7 (DOCX 15 kb) [file 122_2015_2621_MOESM7_ESM.docx]

**Supplementary Table 1**: List of genes selected to develop candidate gene-based markers. The description of the gene functions, literature, and the number of copies found in the diploid species, *B. rapa* and *B. oleracea,* and *B. napus* are provided here as additional information.

|  | Gene | Function | Literature | Gene ID of Arabidopsis | Gene ID of orthologues in *B. rapa*^1^ and B.oleracea^2^ (chromosome) | Gene ID of orthologues in *B. napus* (Chalhoub 2014) |
| --- | --- | --- | --- | --- | --- | --- |
| 1 | *3-hydroxy-3-methylglutaryl CoA reductase 1 (HMG1)* | Encodes a 3-hydroxy-3-methylglutaryl coenzyme A reductase which catalyzes the first committed step in isoprenoid biosynthesis. Generally considered to be a regulatory enzyme in sterol biosynthesis. HMG1 and HMG2 are differentially expressed. | Chye et al. (1992); Enjuto et al. (1994); Harker et al. (2003) | AT1G76490 | Bra008261 (A02) Bra015739 (A07) Bol039236 (C02) Bol027626 (C06) | - BnaA07g32810D BnaC02g24100D BnaC06g37290D |
| 2 | *3-hydroxy-3-methylglutaryl-CoA reductase 2 (HMG2)* |  |  | AT2G17370 | Bra002053 (A10) Bol017681 (C07) | BnaA07g02540D BnaC07g06390D |
| 3 | *Hydroxymethylglutaryl-CoA synthase (HMGS)* | Encodes a 3-Hydroxy-3-methylglutaryl-coenzyme A synthase (HMGS) which catalyzes the condensation of acetoacetyl-CoA and acetyl-CoA to produce S-3-hydroxy-3-methylglutaryl-CoA (HMG-CoA) in mevalonate pathway of isoprenoid biosynthesis. | Wang et al. (2012); Liao et. al. (2014) | AT4G11820 | Bra033126 (A02) Bra014870 (A07) Bol045227 (C06) Bol008312 (UNC)^3^ | BnaA02g21580D BnaA09g21330D BnaCnng45000D BnaC09g23740D |
| 4 | *Sterol methyltransferase 1 (SMT1)* | SMT1 encodes a sterol-C24-methyltransferases which principally methylates cycloartenol into 24-methylene cycloartenol in sterol biosynthesis while SMT2 encodes a sterol-C24-methyltransferases which methylates 24-methylene lophenol into 24-ethyldiene lophenol in sterol biosynthesis. There is some promiscuity in substrate specificity among the SMTs. | Bouvier-Nave et al. (1998); Diener et al, (2000) | AT5G13710 | Bra023430 (A02) Bra006211 (A03) Bol034248 (C03) Bol004256 (UNC) | BnaA02g01810D BnaA03g04460D BnaC03g06000D BnaC02g04950D |
| 5 | *Sterol methyltransferase 2 (SMT2)* |  |  | AT1G20330 | Bra025810 (A06) Bol026945 (C05) | BnaCnng20770D BnaCnng74930D |
| 6 | *Diacylglycerol acyltransferase 1 (DGAT1).* | Encodes Acyl-CoA diacylglycerol acyltransferase (DGAT1) which catalyzes the acyl-CoA-dependent acylation of sn-1,2-diacylglycerol to produce triacylglycerol (TAG). | Li et al. (2010): Liu et al. (2012) | [AT2G19450](https://www.arabidopsis.org/servlets/TairObject?id=32778&type=locus) | Bra036722 (A09)  Bra039003 (A07)  Bol029796 (UNC)  Bol022722 (C07) | BnaAnng30990D BnaA07g36000D BnaCnng52810D - |
| 1. *Brassica rapa* Chiifu gene matches are indicated with prefix "Bra" (Wang 2011) | | | | | | |
| 2. *Brassica oleracea* capitata gene matches are indicated with prefix "Bol" (http://ocri-genomics.org/bolbase) | | | | | | |
| 3. UNC – Not assigned to chromosome | | | | | | |
