# Supplementary material for: Genetic variation and inheritance of phytosterol and oil content in a doubled haploid population derived from the winter oilseed rape Sansibar × Oase cross
Source: Theor Appl Genet. 2015 Oct 30;129:181–99. doi: 10.1007/s00122-015-2621-y (PMC4703628; doi:10.1007/s00122-015-2621-y)
Supplement: Supplementary file 8 — Supplementary material 8 (DOCX 12 kb) [file 122_2015_2621_MOESM8_ESM.docx]

## Supplementary Table 2

Primer sequence of candidate gene-based markers

| Primer name | Primer sequence (5’ 🡪 3’) | |
| --- | --- | --- |
| HMG1A07-O1 | Fwd  Rev | CAGAGGGTGCAAGGCTATGTA  CAAAGAAGCCACGCTCGTC |
| HMG2A10-2 | Fwd  Rev | TGGGACAGTGCTGCGAGA  CCACCAAGAGAACCAGCCATA |
| D120E-3 | Fwd  Rev | CTGCCTTTACCGTCGAGAAAC  AGATCAAGCGGGCAAAAATGG |
| Dx-3 | Fwd  Rev | GTCTTCAGCTAATAGCATCAAACATTC  GTGGAGGGTCAAAGCTAAATTTC |
